# Lewis Structures and the Bonding Classification of End-on Bridging Dinitrogen Transition Metal Complexes

**DOI:** 10.1021/jacs.2c12243

**Published:** 2023-02-16

**Authors:** Faraj Hasanayn, Patrick L. Holland, Alan S. Goldman, Alexander J. M. Miller

**Affiliations:** † Department of Chemistry, American University of Beirut, Beirut 1107 2020, Lebanon; ‡ Department of Chemistry, Yale University, New Haven, Connecticut 06520, United States; § Department of Chemistry and Chemical Biology, Rutgers, The State University of New Jersey, New Brunswick, New Jersey 08903, United States; ∥ Department of Chemistry, University of North Carolina at Chapel Hill, Chapel Hill, North Carolina 27599-3290, United States

## Abstract

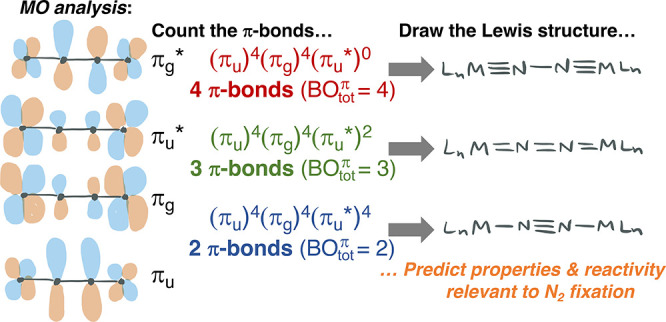

The activation of dinitrogen by coordination to transition metal ions is a widely used and promising approach to the utilization of Earth’s most abundant nitrogen source for chemical synthesis. End-on bridging N_2_ complexes (μ-η^1^:η^1^-N_2_) are key species in nitrogen fixation chemistry, but a lack of consensus on the seemingly simple task of assigning a Lewis structure for such complexes has prevented application of valence electron counting and other tools for understanding and predicting reactivity trends. The Lewis structures of bridging N_2_ complexes have traditionally been determined by comparing the experimentally observed NN distance to the bond lengths of free N_2_, diazene, and hydrazine. We introduce an alternative approach here and argue that the Lewis structure should be assigned based on the total π-bond order in the MNNM core (number of π-bonds), which derives from the character (bonding or antibonding) and occupancy of the delocalized π-symmetry molecular orbitals (π-MOs) in MNNM. To illustrate this approach, the complexes *cis,cis*-[(^iPr4^PONOP)MCl_2_]_2_(μ-N_2_) (M = W, Re, and Os) are examined in detail. Each complex is shown to have a different number of nitrogen–nitrogen and metal–nitrogen π-bonds, indicated as, respectively: W≡N–N≡W, Re=N=N=Re, and Os–N≡N–Os. It follows that each of these Lewis structures represents a distinct class of complexes (diazanyl, diazenyl, and dinitrogen, respectively), in which the μ-N_2_ ligand has a different electron donor number (total of 8e^–^, 6e^–^, or 4e^–^, respectively). We show how this classification can greatly aid in understanding and predicting the properties and reactivity patterns of μ-N_2_ complexes.

## Introduction

Dinitrogen would be an ideal resource in chemical synthesis since it makes up roughly 80% of the Earth’s atmosphere, but this abundant feedstock is resistant to activation and functionalization. The only large-scale industrial reaction of N_2_ commercialized to date is the Haber–Bosch process for ammonia synthesis. While revolutionary in its time,^1^ the Haber–Bosch process employs harsh conditions and relies on hydrogen from fossil fuels, leading to unsustainable levels of CO_2_ emissions. With the demands for ammonia continuing to rise, developing a more sustainable synthesis from N_2_ has become an urgent goal.^2−6^

Chemists working toward N_2_ functionalization under mild conditions have been guided by the hypothesis that binding N_2_ to a transition metal ion can activate it toward synthetic modification.^7^ Numerous metal–N_2_ complexes spanning the periodic table have been prepared, and reactivity of the bound N_2_ ligand has been demonstrated, including catalytic examples of ammonia and hydrazine formation among other reactions.^8−12^

One key factor influencing the prospects and mechanism of functionalization is the N_2_ binding mode,^13^ with end-on terminal (η^1^) and end-on bridging (μ-η^1^:η^1^-N_2_, which we simplify to μ-N_2_ herein) modes most commonly observed. Although it has historically been difficult to predict which mode will be adopted, we recently showed that an MO-based analysis considering the number of d-electrons supplied by the two metals to the π-MNNM manifold can correctly predict the preference for end-on terminal or bridged coordination (eq 1).^14^
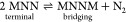
1

Side-on bridging N_2_ coordination is also known, with the nature of the metal and steric factors proposed to be responsible for controlling which bridging mode is adopted;^15−19^ however, we focus on end-on binding here.

**Figure 1:**
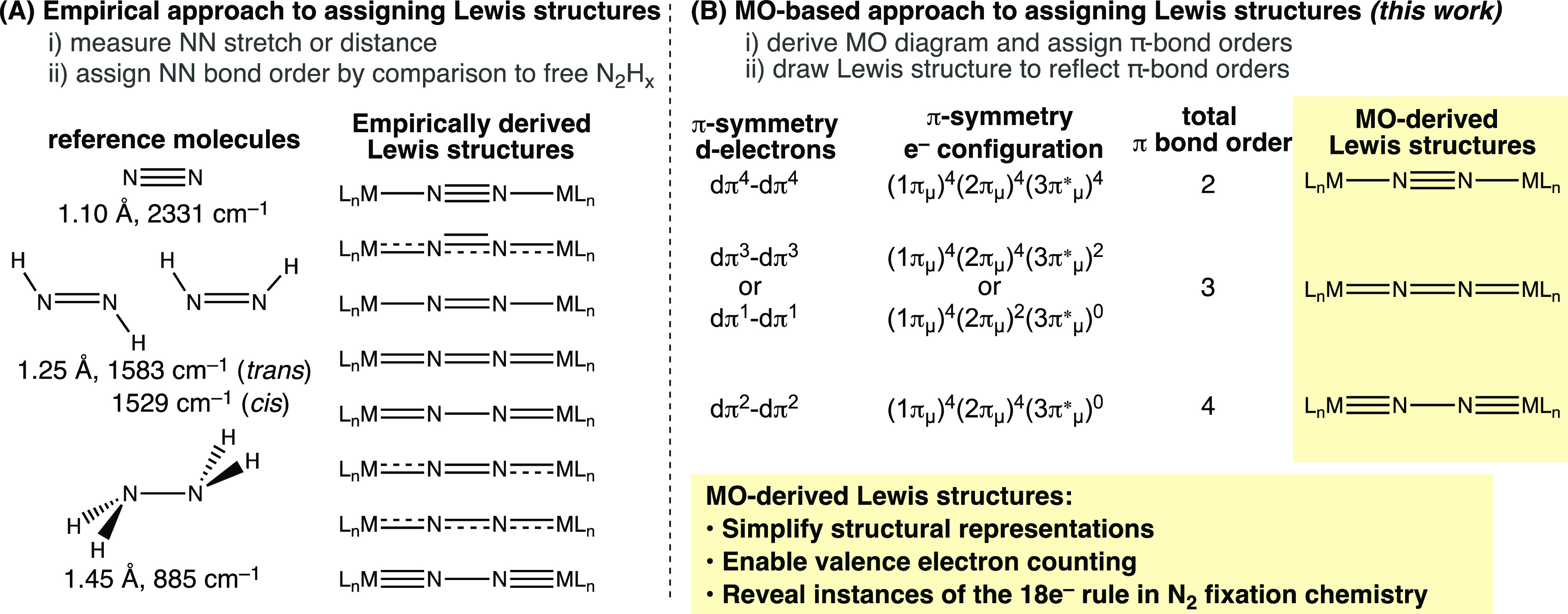
Empirical vs MO-based Lewis structures of the MNNM core of symmetrical μ-N_2_ complexes.

The common view of bonding (and Lewis structure representation) in dinitrogen complexes has been shaped by the synergy model of ligand-to-metal σ-donation and metal-to-ligand π-backdonation,^20−22^ akin to that used for metal–CO bonding^23^ and the original Dewar–Chatt–Duncanson model for η^2^-olefin coordination.^24,25^ Within this model, the degree of π-backbonding upon N_2_ coordination is gauged based on changes in the bond lengths (*r*_NN_) and stretching vibrational frequencies (ν_NN_). For N_2_ binding to a single metal in an end-on mode, crystal structures reveal only minor effects on *r*_NN_, and IR spectroscopy shows similarly small effects on ν_NN_, with the lowest ν_NN_ values reaching around 1910 cm^–1^.^26−29^ The Lewis structure for terminal MN_2_ complexes is therefore generally drawn with a single M–N bond and a triple N≡N bond, M–N≡N. End-on bridged N_2_ coordination, on the other hand, results in changes in *r*_NN_ and ν_NN_ that vary from minimal to substantial, with examples of ν_NN_ even below 1400 cm^–1^, suggesting variations in the NN bond order. This leads to the necessity of making decisions about how to represent the π-bonds in Lewis structures of the MNNM cores, and the prevailing approach is to benchmark against the known values of *r*_NN_ or ν_NN_ in free N_2_, N_2_H_2_, and N_2_H_4_ which are taken as *bona fide* triple, double, and single bonds, respectively.^27,30^ However, many experimental values fall between these benchmarks, leading to ambiguity. Figure 1A illustrates the ambiguity, with drawings from two recent reviews that show *at least eight different Lewis structures* for linear MNNM cores of symmetric, even-electron bridging N_2_ complexes.^31,32^ One could conclude that the large variation in assigning Lewis structures is not indicative of differences in bonding at all, but rather reflects a lack of knowledge about how best to categorize N_2_ complexes into bonding classes.

Lewis structures convey important information about the electronic structure of molecules and communicate their physical properties and chemistry.^33^ A lack of consensus on the appropriate Lewis structure of bridging N_2_ complexes can cloud understanding and discourage comparisons between different complexes even when the MNNM cores might be isoelectronic. Furthermore, without reliable Lewis structures, classic methods for understanding the reactivity of metal complexes such as the valence electron count (VEC) cannot be applied. In chemistry, the 18e^–^ rule provides a simple but powerful tool that in certain contexts enables chemists to predict the coordination number, geometry, and reactivity trends of metal complexes. For example, using the 18e^–^ rule one can correctly predict that the most “stable” binary carbonyl complex of nickel is Ni(CO)_4_, and that H_2_ is likely to undergo oxidative addition to Fe(CO)_4_ but not to Ni(CO)_4_. By assuming ferrocene has an 18e^–^ VEC, Fischer correctly predicted the sandwich structure.^34,35^ Yet, the use of VEC tools has been virtually absent from the literature on μ-N_2_ complexes. We suspect that this arises because it is not clear how to assess the electron donor numbers of some of the Lewis structures from the current N_2_/N_2_H_2_/N_2_H_4_ benchmarking methods.

In the present Perspective, we introduce a general and easily implemented approach to assigning the Lewis structures of end-on bridging N_2_ complexes using the total π-bond order (BO_tot_^π^) of the MNNM core as defined using qualitative π-MO diagrams. To support the validity of the MO approach, we build isolobal analogies to organic molecules.^36^ We begin with a brief review of qualitative MO treatment of simple diatomic, triatomic, and tetratomic molecules, and then extend the same approach to develop a picture of the complexes *cis,cis*-[(^iPr4^PONOP)MCl_2_]_2_(μ-N_2_), [**1**-M]_2_(μ-N_2_), M = W, Re, and Os. We were particularly inspired by the rhenium complex [**1**-Re]_2_(μ-N_2_), which we recently found to be isolable and stable at room temperature, yet still capable of a reaction sequence that converts N_2_ to [NH_4_]^+^.^37^ This series reveals three limiting bonding classifications, each of which has a natural Lewis structure representation of the MNNM core as shown in Figure 1B. An important corollary here is that each drawing represents a distinct μ-N_2_ ligand having a different electron donor number. We argue that the prevailing empirical benchmarking approach to determining Lewis structures is not appropriate because the nitrogen atoms in the common reference molecules diazene and hydrazine are not part of a linear tetratomic core as in the bimetallic N_2_-bridged complexes. Remarkably, applying VEC tools using the MO-based Lewis structures reveals that the given series of [**1**-M]_2_(μ-N_2_) complexes satisfies the 18e^–^ rule for all three metals. Further, the models quickly predict the direction of eq 1 and other reactions. We also show how the MO-based Lewis structures can bring new insights in understanding the splitting reaction of μ-N_2_ complexes into two terminal metal nitride products. Adoption of the new model introduced here promises to advance N_2_ chemistry by enabling systematic comparisons between complexes, accurate predictions of properties and reactivity, and guidelines for the design of new N_2_ complexes.

## π-MO Diagrams and Lewis Structures of N_2_, [N_3_]^−^, and [(NH_3_)_5_Ru(N_2_)]^2+^

In MO theory, delocalized MOs are constructed using linear combinations of atomic orbitals.^38,39^ When symmetry permits, it is possible to construct σ-, π-, or δ-MOs separately. In this Perspective we are mostly interested in π-bonding; we build up starting from simple examples in order to clarify how our model for metal-N_2_ complexes is analogous to these well-accepted molecules.

**Figure 2:**
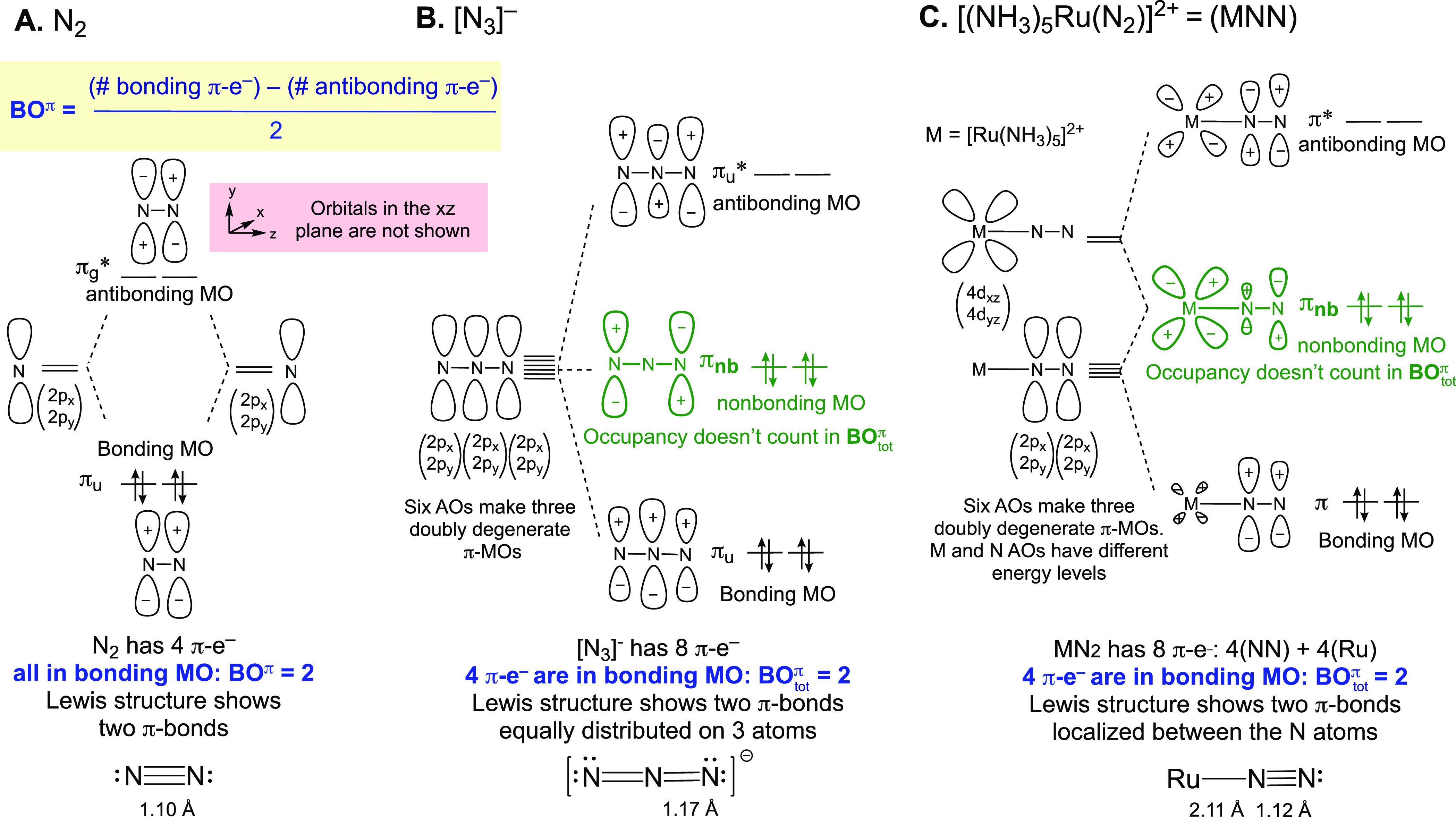
π-MO diagrams and Lewis structures for N_2_, [N_3_]^−^, and [(NH_3_)_5_Ru(N_2_)]^2+^. Only one of each pair of doubly degenerate π-MOs is drawn.

To orient readers with the “π-only” MO diagrams, we first illustrate the diatomic ligand of interest, N_2_ (Figure 2A). The (2p_*x*_,2p_*y*_) AOs of nitrogen are suitable for π-bonding in N_2_, generating a basis set of four AOs. The in-phase combinations of the AOs (constructive interference) create a doubly degenerate bonding π-MO (π_u_) with an energy level lower than that of the AOs. The out-of-phase AO combinations (destructive interference) generate a doubly degenerate antibonding π-MO (π_g_*) with a node between the atoms and an energy higher than that of the AOs. MO diagrams are used to define a π-bond order (BO^π^) as half the difference between the number of electrons in the bonding and antibonding π-MOs (Figure 2A).^40^ For N_2_, each atom provides two electrons to the π-MOs; in the ground state, four electrons fill the low energy bonding π-MO, so BO^π^ = 2. This is equivalent to the number of π-bonds in the Lewis structure of N_2_.

Next, we consider π-bonding in the azide ion [N_3_]^−^ (Figure 2B). In the linear geometry, three pairs of (2p_*x*_,2p_*y*_) AOs are transformed into three delocalized doubly degenerate π-MOs. Estimates of the AO coefficients and relative energies of the π-MOs can be obtained using the Hückel MO method.^41^ In this Perspective we are concerned primarily with the total bond order in linear moieties (BO_tot_^π^). As shown in Figure 2B, the AO interactions in [N_3_]^−^ afford three π-MOs: one bonding (π_u_), one nonbonding (π_nb_), and one antibonding (π_u_*). The characters of these MOs are related to those of the familiar allyl anion and CO_2_, and can be easily obtained by dividing [N_3_]^−^ into two fragments and considering their interactions (Figure SI-1A).^39^ [N_3_]^−^ has 8 π-electrons; four fill π_u_ and four fill π_nb_, resulting in BO_tot_^π^ = 2. Because the terminal atoms are identical, each NN linkage has a “local” NN BO^π^ = 1. This matches the Lewis structure drawn for [N_3_]^−^ based on localized shared electron pairs.

Figure 2C considers π-bonding in the terminal RuNN bond of [(NH_3_)_5_Ru(N_2_)]^2+^, the first reported dinitrogen complex.^42−44^ As in [N_3_]^−^, the (2p_*x*_,2p_*y*_) AOs of nitrogen and the (4d_*xz*_,4d_*yz*_) AOs of Ru are expected to generate three doubly degenerate π-MOs in RuNN. Because the Ru and N AOs have different energy levels, the coefficients from Ru and N will be different in the MOs. In analogy to an ethylene molecule substituted with a π-donor orbital having an energy level higher than the bonding π-MO of ethylene,^45^ we expect the lowest energy π-MO in RuNN to be concentrated on NN, and the second π-MO to have principal coefficients from Ru and the distal nitrogen (Figure SI-2A). Such a nonbonding view of the second π-MO in the MNN bond was adopted by DuBois and Hoffmann to describe the electron density distribution in MNN complexes.^46^

The square pyramidal fragment [(NH_3_)_5_Ru]^2+^ has a d^6^-Ru^II^ center that provides 4 electrons for π-bonding in RuNN. N_2_ provides 4 additional electrons leading to a (π)^4^(π_nb_)^4^ configuration. The BO_tot_^π^ in RuNN is therefore 2, similar to [N_3_]^−^. However, because the bonding π-MO is concentrated on the nitrogen atoms, a Lewis representation of the MO-based BO_tot_^π^ would localize both π-bonds between the nitrogen atoms, Ru–N≡N. The given π-bonding picture in MNN implicates a constant BO_tot_^π^ = 2 independent of the occupancy of π_nb_ and can explain why the NN bond distance of terminal MN_2_ complexes is generally not highly sensitive to the metal. This is fundamentally different from bridging N_2_ complexes, as we will show in the subsequent sections.

## π-MO Diagrams and Lewis Structures of Organic Molecules with Linear Tetratomic Cores

Delocalized π-bonding in organic systems is a classic test of MO theory. We sought similarly simple systems with tetratomic cores that could serve as conceptual “bridges” between familiar organic molecules and transition metal μ-N_2_ complexes.^36^

**Figure 3:**
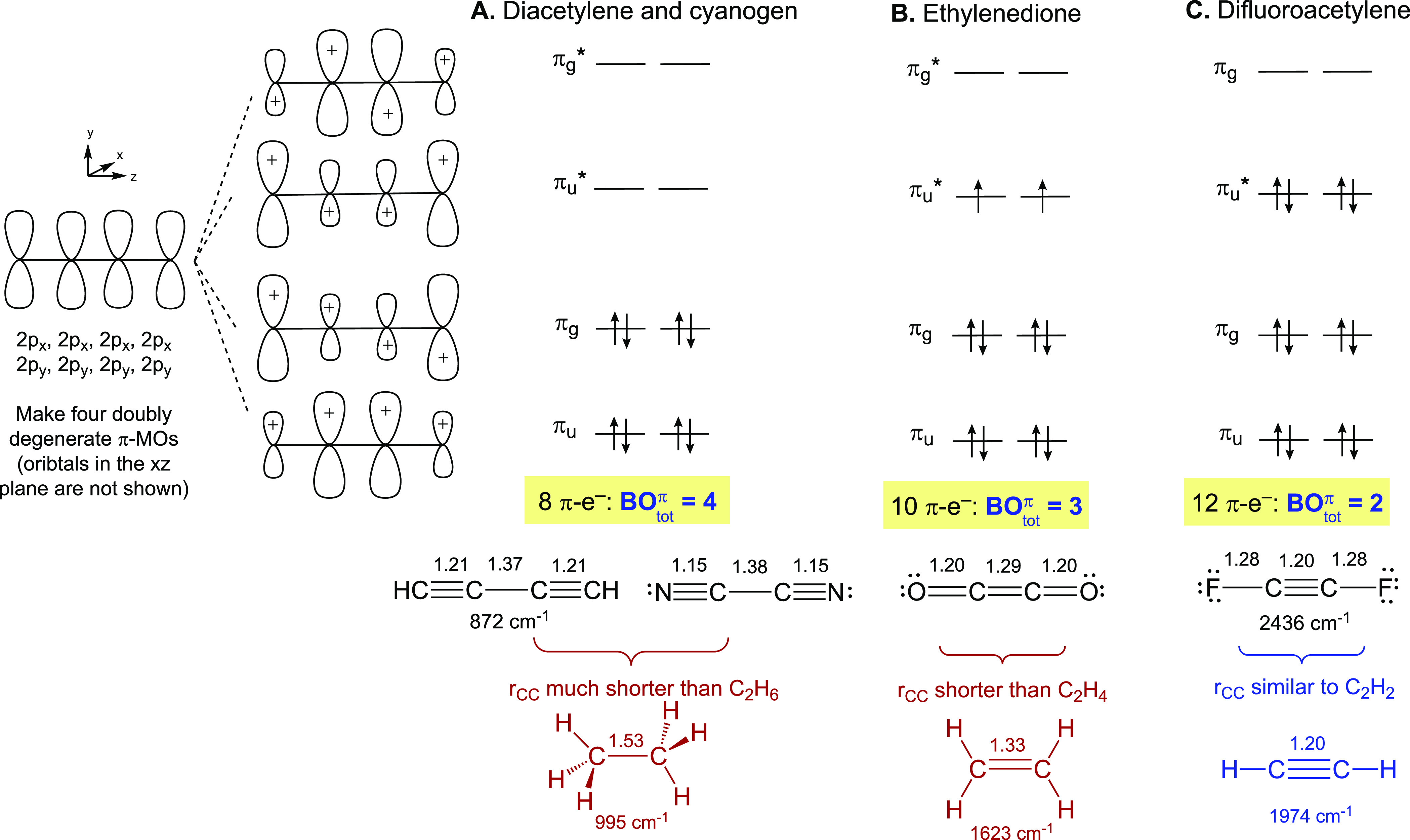
π-MO diagrams and Lewis structures for some linear tetratomic organic molecules. Bond distances in Å. For clarity, only one of each pair of doubly degenerate π-MOs is drawn.

We start in Figure 3 with butadiyne (diacetylene), which has a linear tetratomic core appropriate for building isolobal analogies with the linear MNNM cores of bridging N_2_ complexes. The eight (2p_*x*_,2p_*y*_) AOs of the carbons transform into four doubly degenerate delocalized π-MOs, two bonding (π_u_ and π_g_) and two antibonding (π_u_* and π_g_*). Again, the shape of the MOs can be constructed using the fragment approach (Figure SI-1B), and the AO coefficients can be quantified using the Hückel MO method,^38,41^ or a recently reported graphical method.^47^

Each carbon in diacetylene provides two electrons to the π-system. The total of 8 electrons fills the bonding π_u_ and π_g_, leading to a closed shell singlet state with a total of four π-bonds (BO_tot_^π^ = 4; Figure 3A). Experimentally, the terminal CC bonds in diacetylene are much shorter than the central CC bond, 1.21 versus 1.37 Å.^48,49^ MO theory attributes this difference to the presence of a node in π_g_ that imparts antibonding character selectively to the central CC segment of the MO, thereby removing the “local” π-bond from between the two atoms acquired from the filling of π_u_.^50^ This effect is well captured in the familiar Lewis structure of diacetylene, HC≡C–C≡CH. Consistent with this representation, the bond length of the terminal CC bonds in diacetylene is similar to the C–C bond length in acetylene: 1.21 and 1.20 Å, respectively.^51^ However, although the central carbons in diacetylene, butadiene, and *n*-butane are each represented as being connected by a single σ-bond, the bond length varies considerably among the three molecules: 1.37, 1.47, and 1.53 Å, respectively.^51^ A commonly adopted explanation for these variations is hybridization, with the increased *s*-character in the C–C bond drawing the atoms closer.^38,52,53^ While the detailed contributors to the observed variations can still be a subject of debate,^54−58^ all chemists accept that diacetylene is best represented using a Lewis structure with a central single bond, even though the bond distance is closer to ethylene than it is to ethane (Figure 3). It therefore becomes clear that any bond distance comparisons should take into account whether the bonding picture is similar between the molecules being compared or not.

The MO π-bonding picture in diacetylene is equally applicable to the linear tetratomic species cyanogen, NCCN, which also has 8 π-electrons. The same Lewis structure, N≡C–C≡N, is therefore adopted. Consistently, the CC bond length of cyanogen and the central CC bond in diacetylene are similar.^59^ Note that even though the atoms in cyanogen are not identical, contrasting with the case of the diacetylene core, the π-MOs maintain the same character and phasing; only the orbital coefficients are perturbed due to differences in the electronegativity of the heteroatoms (Figure SI-3). From cyanogen, one can envision a series varying the terminal atoms while moving across the period, from NCCN to OCCO to FCCF. If one considered the CC group a bridge between the terminal atoms, this would be analogous to changing the metal atoms in a MNNM core.

The carbon and oxygen (2p_*x*_,2p_*y*_) AOs in ethylenedione, OCCO, transform into delocalized π-MOs as in diacetylene and cyanogen, but there are now 2 additional electrons in the π-manifold (a total of 10 π-e^–^), so OCCO has a (π_u_)^4^(π_g_)^4^(π_u_*)^2^ configuration.^60^ The partial occupancy of π_u_* *lowers* the total bond order BO_tot_^π^ to 3. Since π_u_* has a π-node in each of the terminal CO bonds but not in CC, the local π-bonding character in the CC bond is *increased* compared to the CC bond in cyanogen, and this is clearly manifested in a significantly shorter CC bond length compared to NCCN, 1.29 versus 1.38 Å. Based on the position of the nodes in the MOs, we arrive at a Lewis structure of O=C=C=O for this molecule.

The use of two parallel arrows in the MO diagram in Figure 3B implies a triplet spin state, but the partial occupancy of π_u_* actually produces three electronic states: a triplet ^3^Σ_g_^–^, a doubly degenerate closed shell singlet ^1^Δ_g_, and an open shell singlet ^1^Σ_g_^+^. From *ab initio* calculations, the ^3^Σ_g_^–^ state is found to be the ground state, with ^1^Δ_g_ and ^1^Σ_g_^+^ higher in energy by 6.9 and 9.5 kcal/mol, respectively.^60^ These states are all computed to have comparable CC bond lengths,^60^ which is expected because they arise from the same MO occupancy, implying BO_tot_^π^ is the same. Note that even though there are two unpaired electrons in the ground state, Lewis structures avoid the use of dashed lines of the types often drawn in analogous MNNM cores.

Lastly, Figure 3C considers difluoroacetylene, FCCF. With 12 electrons in π-symmetry orbitals, the (π_u_)^4^(π_g_)^4^(π_u_*)^4^ configuration gives a closed shell singlet state with BO_tot_^π^ = 2. By the same reasoning based on the position of the nodes followed above, the full occupancy of π_u_* localizes the two π-bonds in between the central carbons. Consistently, FCCF has a shorter CC bond (1.20 Å) than ethylenedione (1.29 Å) and cyanogen (1.38 Å).^61^ The familiar Lewis structure F–C≡C–F accurately captures the electronic and structural features inferred from the π-MOs. Expectedly, the frequency of the CC stretching vibrational mode (ν_CC_) in FCCF (2436 cm^–1^; Figure 3C) is much higher than that of the *C*_2_–*C*_3_ stretching mode of diacetylene (872 cm^–1^; Figure 3A).

The outcome of Figure 3 is a direct connection between the number and position of bonds in Lewis structures and the total bond order and position of the nodes from MO theory. For such organic molecules the Lewis structures are readily drawn without inspection of an MO diagram. But it is clear that assigning Lewis structures for NCCN and OCCO using the CC bond distances or stretch frequencies of ethane and ethylene as reference would not work. This must raise doubts about using diatomic N_2_, N_2_H_2_, and N_2_H_4_ for comparisons to complexes with tetratomic MNNM cores. Drawing Lewis structures directly for multinuclear transition metal complexes can be more challenging, but the task is greatly simplified when one starts with qualitative MO diagrams as we demonstrate in the following section.

## MO-Derived Lewis Structures of μ-N_2_ Complexes

**Figure 4:**
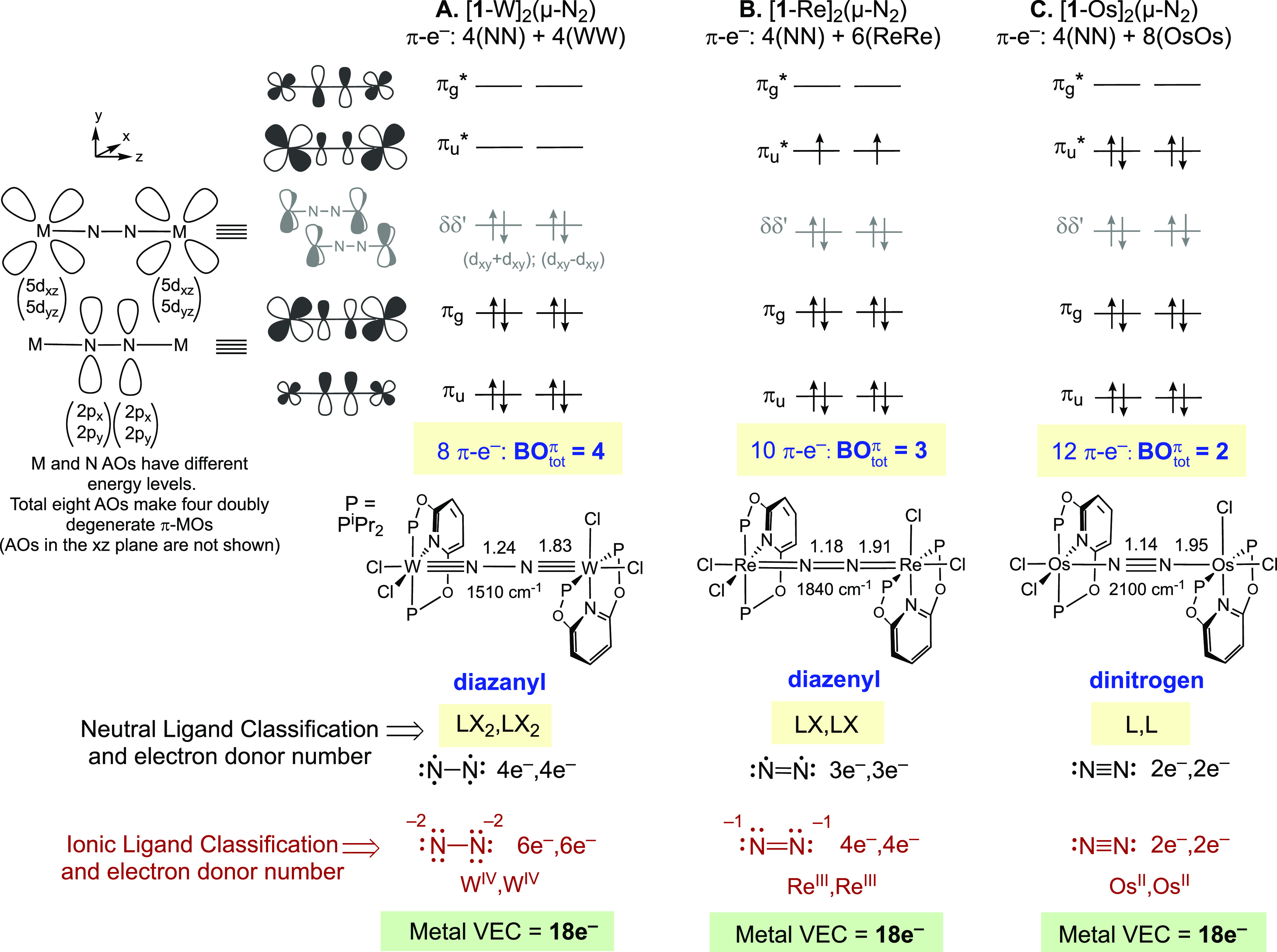
π-MO diagrams, Lewis structures, and Valence Electron Count in [**1**-M]_2_(μ-N_2_) (M = W, Re, and Os). For clarity, only one of each pair of π-MOs is drawn. Note that [**1**-M]_2_(μ-N_2_) has a *C*_2_ point group, but in the pseudo-*D*_4*h*_ geometry the π-MOs (with a and b symmetries) remain essentially degenerate (Figure SI-2). Bond distances in Å.

The π-MO diagrams of the MNNM core of μ-N_2_ complexes can be constructed and analyzed in a fashion analogous to the organic molecules described above in order to inform the systematic drawing of Lewis structures. Figure 4 illustrates the different bonding situations in a series of end-on bridging N_2_ complexes having identical ligands and geometries, *cis,cis*-[(^iPr4^PONOP)MCl_2_]_2_(μ-N_2_), [**1**-M]_2_(μ-N_2_) (M = W, Re, and Os).^37^

The metals in the given series use the (5d_*xz*_,5d_*yz*_) AOs for π-interaction with the (2p_*x*_,2p_*y*_) AOs of nitrogen, forming two bonding MOs, π_u_ and π_g_, and two antibonding MOs, π_u_* and π_g_*, having the same phases (number and position of the nodes) as in the organic tetratomic molecules. The nonbonding 5d_*xy*_ AOs on the two metals transform into a pair of MOs corresponding to their in-phase (d_*xy*_+d_*xy*_) and out-of-phase (d_*xy*_–d_*xy*_) symmetry adapted linear combinations, and are labeled as δδ′ to specify they are orthogonal to the MNNM axis. As noted for cyanogen, the AO coefficients are expected to vary in the different π-MOs of MNNM because nitrogen is more electronegative than M, but this does not alter the bonding/antibonding assignments of the MOs that lead to the calculation of BO_tot_^π^. As we demonstrate in subsequent sections, the π-MO diagram derived in Figure 4 is readily adapted to other geometries in which each metal has two d-AOs with π-symmetry in the MNNM core, but a modified diagram is needed in certain cases such as metallocenes where each metal provides only one d-AO with π-symmetry to the MNNM core.

The two nitrogen atoms provide four electrons to the π-MOs of all μ-N_2_ complexes, so the lowest-energy doubly degenerate MO π_u_ is always filled. The occupancy of π_g_, δδ′, and π_u_*, on the other hand, varies depending on the metal. The fragment [(^iPr4^PONOP)WCl_2_] ([**1-**W]) has a d^4^-W^II^ center. When two d^4^-[**1-**W] metals are bridged with N_2_, four electrons fill the bonding MO π_g_ of WNNW and four electrons fill the nonbonding δδ′. The resulting (π_u_)^4^(π_g_)^4^ π-configuration creates BO_tot_^π^ = 4 in WNNW. Because π_g_ has a node between the N atoms, the bridge is a diazanyl group with the matching Lewis structure of W≡N–N≡W, analogous to diacetylene and cyanogen. Octahedral W and Nb μ-N_2_ complexes with (π_u_)^4^(π_g_)^4^ configurations are known, and have previously been assigned M=N=N=M or M=N–N=M Lewis structures based on the crystallographic NN bond length (*r*_NN_ = 1.22–1.28 Å) and the method outlined in Figure 1A.^62,63^ These examples illustrate how the empirical and the MO-based Lewis structures can often differ: the MO–based Lewis structures would all be assigned M≡N–N≡M. The computed *r*_NN_ in [**1-**W]_2_(μ-N_2_) is 1.24 Å, similar to the experimentally studied complexes—but far shorter than *r*_NN_ in hydrazine (1.45 Å). We have seen earlier, however, how diacetylene and cyanogen exhibit short C–C bonds that are still best represented as single bonds in Lewis structures. Similarly, *comparisons to hydrazine are not helpful in calibrating structural representations in MNNM*.

Next, Figure 4B illustrates the dirhenium complex [**1-**Re]_2_(μ-N_2_). The MNNM core now has 10 π-electrons in a (π_u_)^4^(π_g_)^4^(π_u_*)^2^ configuration leading to BO_tot_^π^ = 3. Just as with ethylenedione, the partial occupancy of π_u_* gives rise to three different electronic states. DFT calculations predict [**1-**Re]_2_(μ-N_2_) to have a triplet spin ground state, and put the open and closed shell singlet states at 9.4 and 11.4 kcal/mol, respectively.^37^ Experimentally, solutions of [**1-**Re]_2_(μ-N_2_) exhibit spectroscopic signatures consistent with temperature-independent paramagnetism (TIP).^37^ Because π_u_* has nodes between the M and N atoms and no node between the N atoms, the bridge is a diazenyl group with the matching Lewis structure of Re=N=N=Re, with two fewer MN π-bonds and one more NN π-bond compared to [**1-**W]_2_(μ-N_2_). The ReNNRe core in [**1-**Re]_2_(μ-N_2_) is isolobal to ethylenedione and thus adopts an analogous Lewis structure. Finally, in the diosmium complex [**1-**Os]_2_(μ-N_2_) shown in Figure 4C there are 12 π-electrons in a (π_u_)^4^(π_g_)^4^(π_u_*)^4^ configuration leading to BO_tot_^π^ = 2 and an Os–N≡N–Os Lewis structure that is analogous to difluoroacetylene, F–C≡C–F.

The trends in the computed *r*_MN_ and *r*_NN_ in the three complexes in Figure 4 are consistent with the MO-derived local MN and NN BO^π^ assignments. Thus, in moving from W to Re to Os: (a) there are fewer MN π-bonds, and *r*_MN_ increases uniformly from 1.83 Å (W≡N) to 1.91 Å (Re=N) and to 1.95 Å (Os–N); (b) there are more NN π-bonds, and *r*_NN_ decreases from 1.24 Å (WN–NW) to 1.18 Å (ReN=NRe) and to 1.14 Å (OsN≡NOs) while concomitantly ν_NN_ increases from 1510 to 1840 and to 2100 cm^–1^.^19^ In contrast, focusing only on metal-to-ligand backbonding could have led one to predict the opposite trends in *r*_NN_ and ν_NN_, since one might have expected increased backbonding, shorter *r*_NN_, and lower ν_NN_ for the metals that have more π-electrons. The small magnitudes of the changes in *r*_NN_ for the varied local NN BO^π^ in Figure 4 are in line with the small changes in *r*_CC_ for the linear organic molecules in Figure 3. As we now would expect based on the organic molecules introduced above, the N–N distances in linear N_2_-bridged complexes are not expected to approach the N–N distance of hydrazine (1.45 Å, Figure 1).

## Valence Electron Count (VEC) and the 18e^–^ Rule

With the availability of the MO-based Lewis structures, we can now determine the valence electron count (VEC) on each metal of μ-N_2_ complexes. In Figure 4 we show the electron donor type of a bridging N_2_ in each of the different Lewis structures using both the covalent bond classification (CBC) approach (neutral ligand formalism),^64−66^ and the ionic ligand formalism.

In CBC, μ-N_2_ is removed from each complex as a neutral ligand preserving the number of NN π-bonds in a given Lewis structure. In all cases, each end of the NN bridge acts as an L-type 2e^–^ σ-donor ligand to one metal. Each unpaired electron left on a given N acts as an additional X-type 1e^–^ π-donor to one metal. In the ionic model, on the other hand, enough electrons are added to μ-N_2_ so that each MN π-bond in the Lewis structure is formed by a 2e^–^ dative N to M bond. In [**1-**W]_2_(μ-N_2_), the Lewis structure is W≡N–N≡W, so the neutral NN bridge acts as an LX_2_,LX_2_ ligand, donating two σ-electrons and two π-electrons (total 4 electrons) to each metal. In [**1-**Re]_2_(μ-N_2_), the Lewis structure is Re=N=N=Re and μ-N_2_ acts as an LX,LX ligand, donating 3 electrons to each metal. Finally, for [**1-**Os]_2_(μ-N_2_), there are no MN π-bonds, so μ-N_2_ is an L-type 2e^–^ σ-donor to each metal.

Using the ionic model, the bridging NN in [**1-**W]_2_(μ-N_2_), [**1-**Re]_2_(μ-N_2_), and [**1-**Os]_2_(μ-N_2_) would correspond to hydrazine-tetraide ([N_2_]^4–^), diazene-diide ([N_2_]^2–^), and dinitrogen (N_2_) respectively, and the metals would have varied formal oxidation states: W^IV^, Re^III^, and Os^II^. Strikingly, therefore, the MO-based analysis leads to Lewis structures in which the bridging N_2_ ligand alters its donor number depending on the metal, leading thereby to a constant VEC = 18e^–^ across the series. Note that any Lewis structure other than the one used for each specific complex in Figure 4 would have resulted in a VEC ≠ 18e^–^. Only with a systematically applied Lewis structure can a meaningful valence electron count be performed. The variable electron donor properties of the N_2_ bridge have similarities to other ligands such as the terminal nitrosyl and oxo ligands.^65,66^

**Scheme 1:**
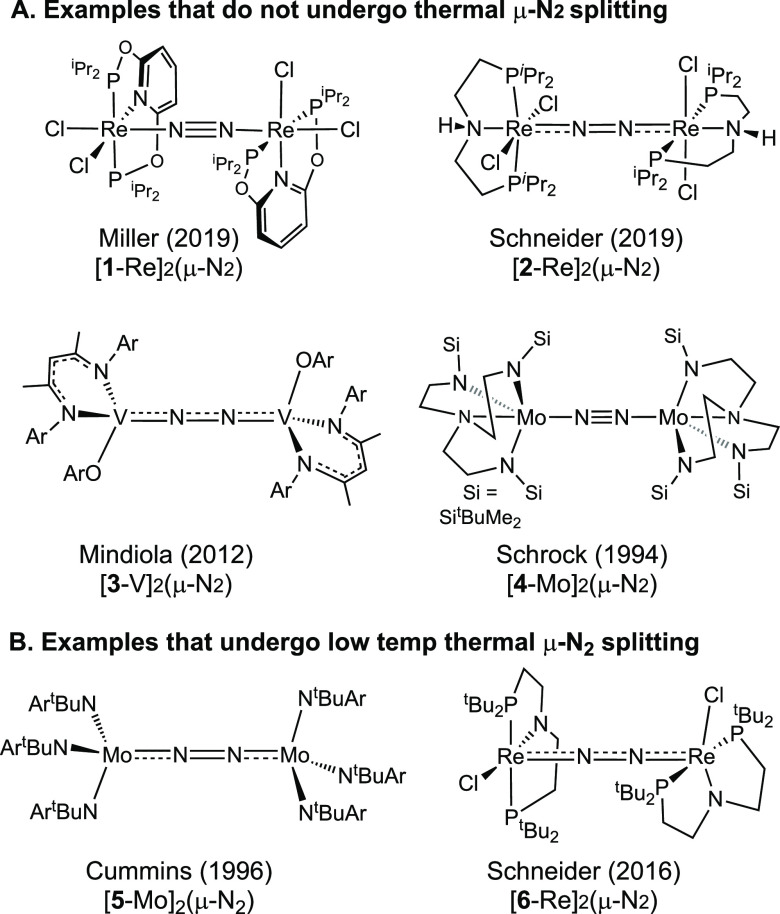
Representative Isolable μ-N_2_ Complexes Having 10 π-e^–^ M=N=N=M Cores Lewis structures are given as originally reported in the literature.

Establishing that the complexes in Figure 4 have filled valences (i.e., having 18 valence electrons) reveals some interesting similarities to the organic molecules discussed in Figure 4 as well as to the diatomic molecules N_2_, O_2_, and F_2_. In particular, [**1-**Re]_2_(μ-N_2_) is a persistent diradical with a full valence shell, similar to that encountered in O_2_ and ethylenedione. Linear tetratomic organic molecules with 10 π-electrons have been of great interest but proved to be challenging to make or even detect. Ethylenedione, for example, is predicted to be intrinsically unstable toward dissociation into two CO molecules,^67^ and has never been experimentally detected, while the isoelectronic ethylenedithione SCCS can be prepared and characterized only at low pressures.^68^ [**1-**Re]_2_(μ-N_2_) therefore provides a striking example of an isolable system with 10 π-electrons in a linear tetratomic core. Other examples of isolable and crystallographically determined 10 π-e^–^ μ-N_2_ complexes are given in Scheme 1, including two that undergo facile (*below* room temperature) cleavage into two terminal nitrides, a reaction of immense current interest in the chemistry of N_2_ functionalization.^32,69^

As shown in Scheme 1, various Lewis structures have been reported for the isolated 10 π-e^–^ complexes, typically using dashed lines. But the MO-derived method assigns the same Lewis structures for all of these, namely M=N=N=M. Once we recognize that this family of N_2_-splitting complexes all have the same Lewis structures, we can start to see how the three distinct Lewis structures with varied numbers of π-bonds correlate with distinct reactivity patterns, which we follow up on in the following sections.

## Lewis Structure and the Reactions of [1-M]_2_(μ-N_2_) with N_2_ and H_2_

**Figure 5:**
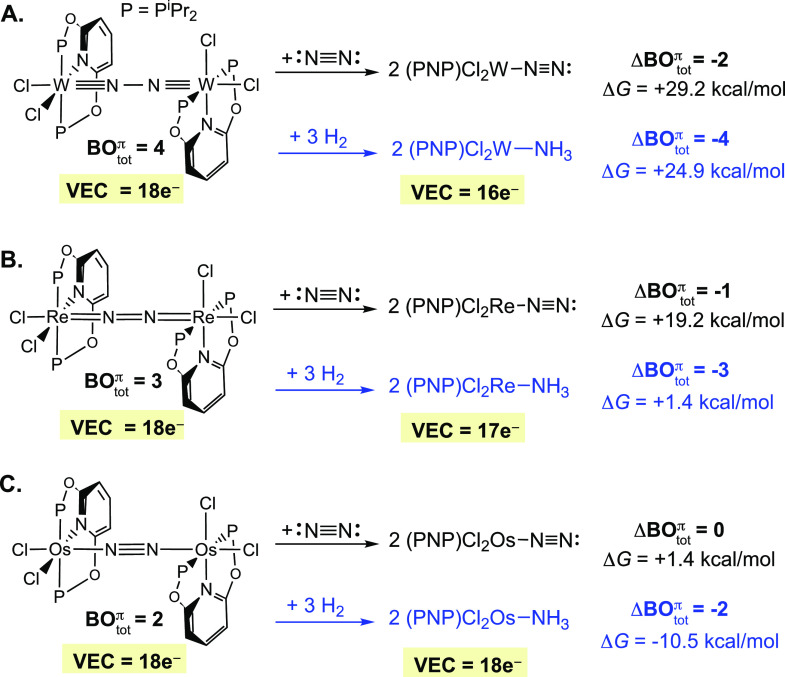
ΔBO_tot_^π^ and energy trends in the reaction of [**1-**M]_2_(μ-N_2_) with N_2_ and H_2_.

To illustrate how categorizing bridging N_2_ complexes based on the MO-derived Lewis structures can aid in predicting thermodynamic trends we consider in Figure 5 the reaction between [**1-**M]_2_(μ-N_2_) and N_2_ to give two octahedral terminal MN_2_ products (L_5_M-N_2_). In the given reaction, the number of σ-bonds is the same in the reactants and the products, so we can limit our analysis to the π-bonds.

As discussed in Figure 2, the terminal MNN moiety in the product has a fixed BO_tot_^π^ = 2 regardless of the number of d_π_ electrons provided by the metal, and thus the Lewis structure is uniformly represented as M–N≡N. The BO^π^ in the free N_2_ molecule is also 2. Because BO_tot_^π^ in the MNNM core of [**1-**M]_2_(μ-N_2_) varies, the transformation from bridging to terminal coordination is associated with a change in the total number of π-bonds (ΔBO_tot_^π^) that varies depending on the metal. The diazanyl complex [**1-**W]_2_(μ-N_2_) has BO_tot_^π^ = 4; when it reacts with N_2_ to form two terminal N_2_ complexes, two π-bonds are lost (ΔBO_tot_^π^ = −2) so the reaction is expected to be highly unfavorable. The analogous reaction of the diazenyl complex [**1-**Re]_2_(μ-N_2_) results in the loss of only one π-bond (ΔBO_tot_^π^ = −1), and it too is expected to be disfavored, but not as much as when ΔBO_tot_^π^ = −2. In contrast, in the reaction of [**1-**Os]_2_(μ-N_2_) with N_2_ no bonds are gained or lost (ΔBO_tot_^π^ = 0), so the reaction is expected to be nearly ergoneutral. In the latter case, attractive dispersion interactions can play a role in favoring bridge formation.^70,71^ The computed free energies included in Figure 5 strongly support the qualitative conclusions based on ΔBO_tot_^π^.^14^

Another way to use the Lewis structures to explain the reaction energy trends in Figure 5 is to compare the VECs in the reactants and products. Regardless of the initial donor number, when the N_2_ ligand converts from bridging to terminal coordination it becomes a simple 2e^–^ L type ligand. Note that N_2_ is a 2e^–^ donor even in the limiting M=N=N Lewis structure of a terminal M–NN bond, acting as an X_2_ type ligand. As such, the different classes of bridging 18e^–^ complexes yield terminal MNN complexes having variable VECs. One anticipates that the bridging complexes with two 18e^–^ metal centers, [**1-**W]_2_(μ-N_2_) or [**1-**Re]_2_(μ-N_2_), should be favored over the corresponding monometallic terminal N_2_ complexes that have VECs of 16e^–^ and 17e^–^, respectively. On the other hand, [**1-**Os]_2_(μ-N_2_) yields terminal MNN products with VEC = 18e^–^ so there is, *a priori*, no reason to expect one side of the reaction to be favored over the other.

As a variation on the theme of how the MO-based Lewis structures can be used to predict reaction energy trends, Figure 5 also considers the hydrogenation of [**1-**M]_2_(μ-N_2_) into two monomeric octahedral ammine complexes using three H_2_ molecules. For the different metals, the same reaction is associated with a different change in the number of π-bonds: ΔBO_tot_^π^ = −4 (W), −3 (Re), and −2 (Os), so one would expect increasingly exergonic reactions across the series. This has been confirmed using new calculations, which afford: Δ*G* = +24.9 (W), +1.4 (Re), and −10.5 (Os) kcal/mol.^72^ We see here that this transformation, hydrogenation of the bridging N_2_ ligand, is counterintuitively *much more exergonic* for the complexes *having N*_*2*_*bonds that are less “activated”*.

## Lewis Structure and the Splitting of μ-N_2_ Complexes

The splitting of bridging N_2_ into two nitrides is one of the most intriguing reactions in transition metal chemistry. The reaction was first discovered by Laplaza and Cummins in 1995 for [**5**-Mo]_2_(μ-N_2_) (Scheme 1B) upon treating the isolable three-coordinate fragment Mo(N^t^BuAr)_3_ with N_2_.^73^ The reaction is of immense interest because it suggests distinct pathways in catalytic N_2_ reduction and functionalization.^32,69,74−80^ In this section we show how Lewis structures can greatly simplify the discussion of this reaction, and even bring new insights to understanding it. Instead of continuing our presentation with the octahedral complex [**1-**Re]_2_(μ-N_2_) which undergoes NN cleavage photolytically but not thermally, we chose to study Cummins’ original complex [**5**-Mo]_2_(μ-N_2_).

**Figure 6:**
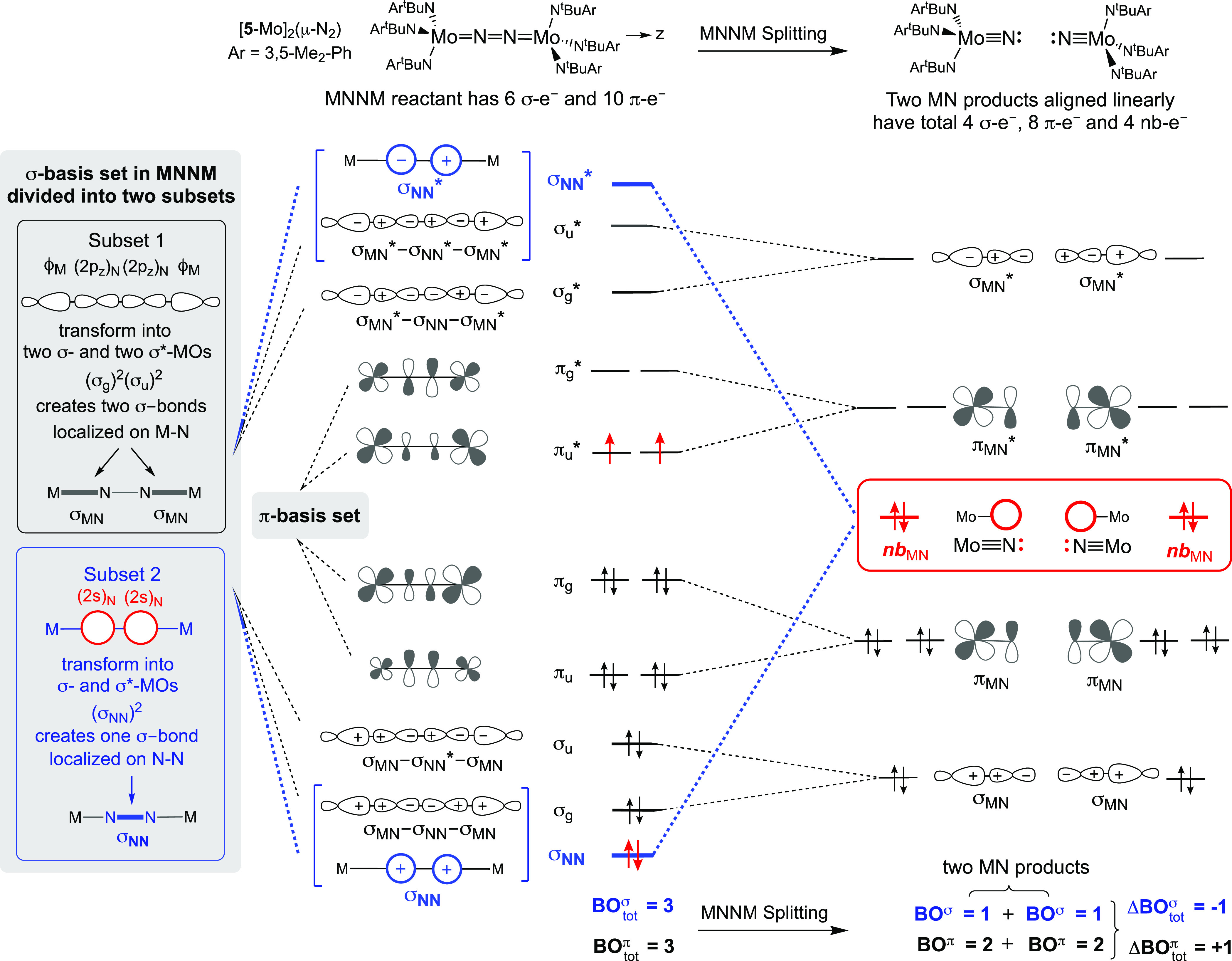
Orbital correlation diagram for splitting of [**5**-Mo]_2_(μ-N_2_) meant to clarify the nature of the σ- and π-MOs in the reactants and products; the aggregation of the π- and π*-MOs above the σ and below the σ* ones is arbitrary. The δδ′-MOs derived from (d_*xy*_,d_*x*^2^–y^2^_) have antibonding character in this system and are omitted. The π-MOs are doubly degenerate, but only one MO is drawn.

In order to discuss the splitting reaction, it is essential to have an orbital correlation diagram that tracks both the σ- and π-bonds in the reactants and products, which we present in Figure 6. Orbital correlation diagrams for this system were previously reported for [**5**-Mo]_2_(μ-N_2_),^69,81,82^ and have also proved useful in studies of metal-mediated oxygen atom transfer reactions.^83^ Our own approach to the same type of diagram is detailed in this section. The (3d_*xz*_,3d_*yz*_) AOs of each metal in [**5**-Mo]_2_(μ-N_2_) have the right symmetry and orientation to undergo π-interactions with the (2p_*x*_,2p_*y*_) AOs of the bridging nitrogen, leading to the same qualitative π-MO diagram of the octahedral complexes in Figure 4. To construct the σ-MOs, we assume that the total σ-bonding in MNNM derives from one empty hybrid AO (ϕ) from each metal and the full (2s,2p_*z*_) AOs from each nitrogen (total six AOs). To simplify the problem, we divide these into two subsets as shown on the left side of Figure 6. Subset 1 includes the two metal hybrid AOs and the two 2p_*z*_ AOs of nitrogen. Subset 2 includes the 2s AOs of nitrogen.

Subset 1 generates two bonding (σ_g_ and σ_u_) and two antibonding (σ_g_* and σ_u_*) MOs delocalized over MNNM. Subset 2 generates two MOs localized on the nitrogen atoms and accordingly labeled as σ_NN_ and σ_NN_*. The MOs from the two sets that have the same symmetry are combined in brackets, [σ_NN_,σ_g_] and [σ_u_*,σ_NN_*], to indicate that these can mix, but that such mixing does not change the overall bonding/antibonding classification of the MOs. An important result from this analysis is that none of the unoccupied antibonding σ-type MOs have bonding character in M–N; σ_g_* and σ_u_* each have a node intersecting the M and N atoms. The σ- and π-MOs can now be combined. The number and character of the MOs is critical in developing a generic correlation diagram for MNNM splitting, but the precise energy level ordering is not consequential. We find it helpful to aggregate the π- and π*-MOs above the σ and below the σ* ones. Note that, for this specific complex, the δδ′-MOs derived from (d_*xy*_,d_*x*^2^–*y*^2^_) are omitted from the figure because these acquire high antibonding character due to mixing with the amido ligands.

The symmetry of the MOs in the correlation diagram can be preserved by aligning the MN bonds of the two nitride products linearly as shown at the right of Figure 6. This makes it clear that MOs σ_g_, σ_u_, π_u_, and π_g_ of MNNM, which are bonding in M–N, correlate with the two bonding σ_MN_ MOs and the two bonding π_MN_ MOs of the products. On the other hand, MOs π_u_*, π_g_*, σ_g_*, and σ_u_* of MNNM, which are antibonding in M–N, correlate with the antibonding MOs σ_MN_* and π_MN_* of the nitrides. Lastly, and perhaps most importantly for our purposes, Figure 6 shows without ambiguity that MOs σ_NN_ and σ_NN_* of MNNM correlate with two *nonbonding* MOs in the MN products (***nb***_MN_).

Figure 6 provides an opportunity to analyze how the bond order changes during N_2_ splitting. [**5**-Mo]_2_(μ-N_2_) has 6 σ-electrons in (σ_NN_)^2^(σ_g_)^2^(σ_u_)^2^ configuration leading to ΔBO_tot_^σ^ = 3. Population of σ_NN_ generates a bond localized between the two N atoms, and population of σ_g_ and σ_u_ generates two σ-bonds localized between the MN atoms (σ_MN_). The π-system in this μ-N_2_ complex has 10 electrons in (π_u_)^4^(π_g_)^4^(π_u_*)^2^ configuration leading to a ground state with BO_tot_^π^ = 3 and two unpaired electrons, with one local π-bond in each MN and NN. The σ- and π-bonds add up to a Mo=N=N=Mo Lewis structure. [**5**-Mo]_2_(μ-N_2_) is experimentally characterized to have a triplet spin ground state.^22^

**Scheme 2:**
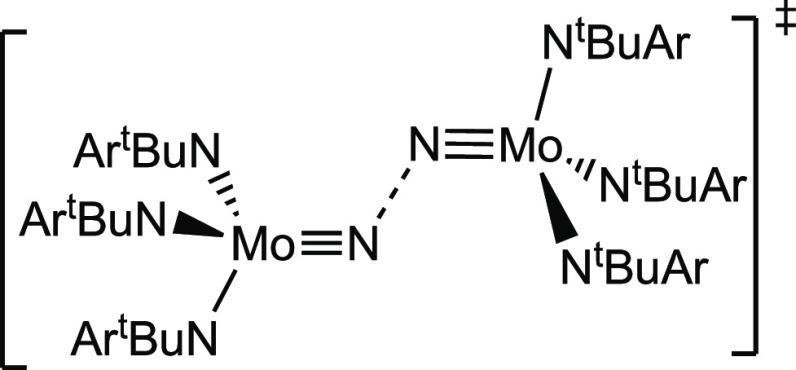
Transition State Structure for MNNM Splitting

To achieve the splitting reaction of Figure 6, an electronic rearrangement within [**5-**Mo]_2_(μ-N_2_) can be envisioned that moves the two unpaired electrons from the doubly degenerate π_u_* into σ_NN_*. Emptying π_u_* reduces the local NN BO^π^ to 0 and increases each local MN BO^π^ to 2. Filling σ_NN_* also drops BO^σ^ to 0; the resulting (σ_NN_)^2^(σ_NN_*)^2^ configuration indicates the electrons reside in effectively nonbonding orbitals that can be regarded as lone pairs (***nb***_MN_). The combined effects lead to a [Mo≡N::N≡Mo] Lewis structure—two separated metal nitride complexes.^84^ The given reaction is symmetry and spin forbidden. Theoretical studies support a reaction pathway for splitting taking place via a trans bending mode of the MNNM core (zigzag transition state structure; Scheme 2) that relieves the orthogonality of the π- and σ-MOs from one plane of the linear structure.^85^ In this specific system, rotation of the amido ligands was proposed to aid reaching the splitting transition state.^86^

The general features of Figure 6, including the requirement of intersystem crossing, align with other reports on the mechanism of MNNM splitting.^22,69,81,82^ Our description does include a few subtle but important differences, however. Previous diagrams relied on using symmetry labels to correlate the MOs in the reactants, the bent transition state, and the products, but did not show the lone electron pairs of the nitride products. Instead, they matched a σ*-MO in the μ-N_2_ reactant with a bonding σ-MO in the nitride products suggesting the *creation of one new MN σ-bond*—but this cannot be correct! Figure 6 shows that MNNM splitting *breaks one NN σ-bond*. The discrepancy arises because the σ*-MO in the commonly used correlation diagrams had been presented as having bonding character in MN and antibonding character in NN,^22^ often labeled as σ_MN_–σ_NN_*−σ_MN_.^69,81,82^Figure 6 shows that the σ*-MOs of MNNM cannot have MN σ-bonding character. The σ_MN_–σ_NN_*−σ_MN_ MO is the same filled bonding σ_u_ which contributes to making the M–N σ-bonds in MNNM; these bonds are retained in the products. The MO-based Lewis structures of [**5-**Mo]_2_(μ-N_2_) and the nitride product of cleavage also align with the bonding changes described in Figure 6. The N_2_-bridged Lewis structure has three σ-bonds and three π-bonds across the MNNM core, while the two nitride complexes have one σ-bond and two π-bonds each, consistent with loss of a σ–σ bond and formation of one π-bond during splitting.

Ethylenedione provides a direct isolobal analogy with an organic system that is also linear as well as has a triplet ground state and an O=C=C=O Lewis structure (Figure 3). An elaborate CASSCF *ab initio* study by Gordon and co-workers taking into account spin–orbit coupling interactions showed this molecule to undergo splitting following intersystem crossing via a zigzag transition state having a geometry remarkably similar to the one reported for MNNM.^60^ A quasi atomic orbital (QUAO) analysis provided a visual representation of how the CC MOs of the reactant evolve into two partially occupied carbon lone pairs in the transition state, and how two lone pairs eventually buildup at the carbons of the separated products, [O≡C: :C≡O]).^60^ This analysis is in full accord with the one presented in Figure 6 for MNNM splitting.

The MO view of the splitting reaction based on Figure 6 would be equally applicable to any end-on bridging N_2_ complex with 10 π-electrons in an M=N=N=M Lewis structure. In fact, the important N_2_ splitting reactivity has been reported only for complexes with BO_tot_^π^ = 3, although the examples in Scheme 1A clearly indicate a 10 π-e^–^ configuration alone is not sufficient for splitting. Using the MO-based Lewis structures to count the number of bonds and valence electrons provides a good starting point to discuss some of the observed reactivity patterns.

**Figure 7:**
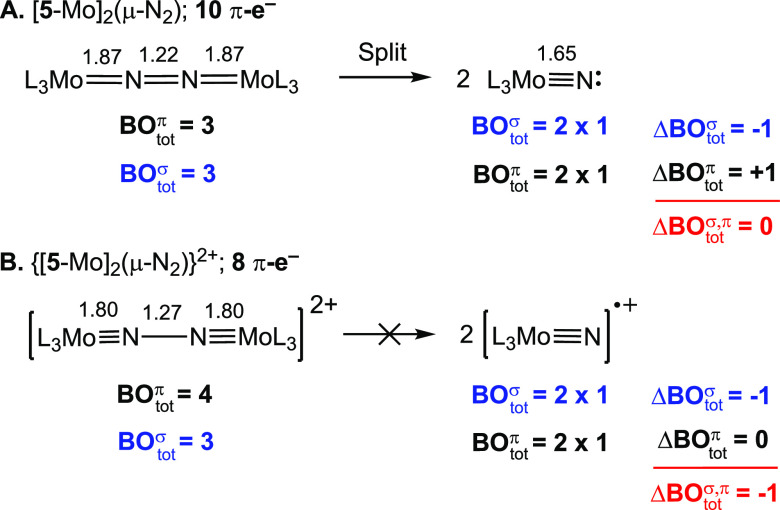
Change in the number of bonds upon splitting of [**5**-Mo]_2_(μ-N_2_) and its dication. Crystallographic bond lengths in Å.

Figure 7 compares the change in the total σ- and π-bond orders (ΔBO_tot_^*π,σ*^) upon splitting of [**5**-Mo]_2_(μ-N_2_) and its dicationic congener, which is also known and crystallographically characterized.^86^ The neutral complex has a Mo=N=N=Mo Lewis structure; when it splits into two neutral nitrides Mo≡N:, the NN σ-bond is cleaved and a new π-bond is formed, amounting to ΔBO_tot_^*π,σ*^ = 0. The bridging dication, on the other hand, has 8 π-e^–^ in a (π_u_)^4^(π_g_)^4^ configuration yielding BO_tot_^π^ = 4 and an [Mo≡N–N≡Mo]^2+^ Lewis structure. Despite the increasingly stretched NN bond and the double positive charge that one might anticipate to favor cleavage from a purely electrostatic perspective, this dication is stable at 20 °C both as a solid and in solution. NN splitting in this case breaks one σ-NN bond (ΔBO_tot_^σ^ = −1) without a change in the number of MN π-bonds (ΔBO_tot_^π^ = 0). Based on the net loss of a bond (ΔBO_tot_^*σ,π*^ = −1) one anticipates the reaction to be less exothermic than the splitting of the neutral [**5**-Mo]_2_(μ-N_2_).

In closing this section, we consider how factors beyond the bond order, such as the attributes of the bonds or effects from the ancillary ligands, can affect N_2_ splitting reactivity. The fundamental principles that quantitatively govern the splitting of 10 π-electron M=N=N=M complexes are still being elucidated, but at least two additional factors beyond change in the number of bonds can influence the reaction. First, the 4d and 5d transition metals have strong capacity for MN π-bonding due to relativistic effects,^87^ and thus the increase in the number of π-bonds upon splitting is particularly beneficial in terms of energetics. In the absence of such effects in 3d metals, loss of σ_NN_ is not well compensated for by the gain in π_MN_. Accordingly, the vanadium complex [**3**-V]_2_(μ-N_2_) in Scheme 1A does not split even though it is structurally and electronically similar to [**5**-Mo]_2_(μ-N_2_).^88^ Second, the splitting energies are highly dependent on the presence or absence of a ligand *trans* to the bridging N_2_. For example, the 10 π-e^–^ five-coordinate trigonal bipyramidal complex [**4**-Mo]_2_(μ-N_2_) in Scheme 1 has a 4d metal yet is not reported to undergo thermal splitting, in contrast to the facile cleavage of the closely related four-coordinate tetrahedral [**5**-Mo]_2_(μ-N_2_).^89^ Likewise, thermal NN splitting has been observed for five-coordinate 10 π-e^–^ complexes in square pyramidal geometries that leave a vacant coordination site *trans* to the N_2_ bridge, as exemplified by Schneider’s complex [**6**-Re]_2_(μ-N_2_) shown in Scheme 1B,^90,91^ but not for the octahedral complexes [**1**-Re]_2_(μ-N_2_) and [**2**-Re]_2_(μ-N_2_) in Scheme 1A.^81^ The nitride ligand has a very strong *trans* influence (induced by the exceptionally short MN bond length);^92^ thus N–N splitting is disfavored by the presence of a trans ligand.

## Assigning MO-Derived Lewis Structures for Other Geometries

**Figure 8:**
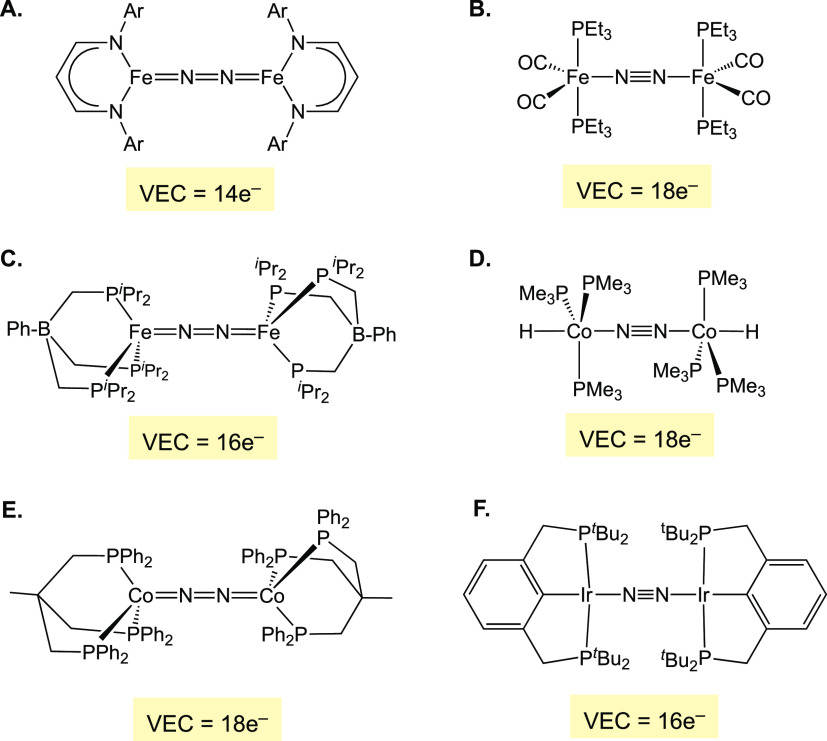
MO-based Lewis structures and VECs of selected μ-N_2_ complexes with different geometries.

So far, we have focused on symmetrical octahedral and tetragonal μ-N_2_ complexes. In our prior work, we assigned BO_tot_^π^ for 25 complexes ranging from three- to six-coordinate and covering 3d, 4d, and 5d transition metals.^14^ Representative examples are shown in Figure 8. The π-bonding picture was established using DFT methods coupled to experimental spectroscopic data. Qualitative MO diagrams for some of the geometries are given in Figures SI-4–6 of this Perspective. In the following sections, we discuss some aspects of high-spin complexes, and we assign Lewis structures to two unique classes of complexes that we did not consider before, namely heterobimetallic complexes and metallocenes.

## High Spin Complexes

A prototype of a high-spin μ-N_2_ complex is the three-coordinate diiron(I) complex (Figure 8A) with a septet spin ground state.^93^ This complex has a 10 π-e^–^ configuration, so it is assigned an M=N=N=M Lewis structure leading to a 14e^–^ VEC. The given Lewis assignment agrees with the experimentally observed substantial weakening of the NN bond that is much greater than in the lower-valent phosphine-carbonyl iron complex (Figure 8B).^94^ For another example, we return to the octahedral complex [**1-**W]_2_(μ-N_2_). We discussed above (Figure 4) the singlet state of this complex from the (π_u_)^4^(π_g_)^4^(δδ′)^4^ configuration where each metal provides 2e^–^ to the π-MOs and 2e^–^ to δδ′. Alternatively, each metal may provide 3e^–^ to the π-manifold and only 1e^–^ to δδ′ leading to a (π_u_)^4^(π_g_)^4^(δδ′)^2^(π_u_*)^2^ configuration. The additional partial occupancy of π_u_* lowers BO^π^ to 3, so the Lewis structure would be W=N=N=W. Consistently, the computed quintet state of [**1-**W]_2_(μ-N_2_) has longer WN (1.93 Å) and shorter NN (1.19 Å) bonds than the singlet state (1.83 and 1.24 Å respectively), such that the quintet state is very similar to the related 10 π-e^–^ species [**1-**Re]_2_(μ-N_2_).^14^ Despite the lower ΔBO_tot_^π^, the computed energy of the quintet spin state of [**1-**W]_2_(μ-N_2_) is only 8.5 kcal/mol higher than the closed shell singlet, suggesting similar energies associated with pairing two electrons in δδ′ or promoting two electrons to π_u_*. Indeed, examples of d^4^–d^4^ μ-N_2_ complexes with open-shell ground states are known,^95,96^ and a spin-state change had been proposed to initiate N_2_ splitting.^95^ The model can therefore be valuable to predict differences in properties or reactivities as a function of spin state.

## Heterobimetallic μ-N_2_ Complexes

**Figure 9:**
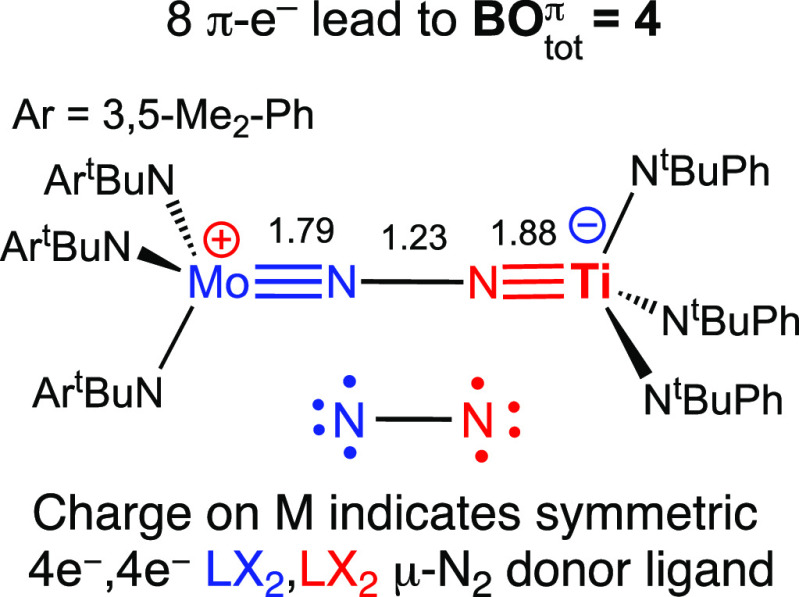
Lewis structure for the heterobimetallic complex [**5-**MoTi](μ-N_2_). Crystallographic bond distances in Å.

While qualitative π-MO diagrams can extract a BO_tot_^π^ term for known heterobimetallic μ-N_2_ complexes,^97−102^ some systems require Lewis structure representations distinct from the ones used for the symmetrical ones. For an illustrative example, we consider in Figure 9 the mixed Mo/Ti complex [**5-**MoTi](μ-N_2_),^103^ the direct analogue of the symmetrical [**5-**Mo]_2_(μ-N_2_) discussed above. The Mo/Ti complex is characterized by short MoN (1.79 Å) and TiN (1.88 Å) bonds, a significantly stretched NN bond (1.23 Å), and a quite low ν_NN_ (1575 cm^–1^).^103^

Starting with the same MO diagram of [**5-**Mo]_2_(μ-N_2_) derived in Figure 6 and then removing two electrons to account for titanium having two less electrons than Mo leads to a (1π)^4^(2π)^4^ configuration (the different metals prevents the use of π_u_ and π_g_ labels for the π-MOs in this case). The MoNNTi core therefore has BO_tot_^π^ = 4 with two π-bonds in each of MoN and TiN and none in NN, fully consistent with the experimental parameters. However, merely using the Mo≡N–N≡Ti Lewis structure leads to unreasonable formal oxidation states, Mo^V^ and Ti^V^, and unreasonable VECs.^104^ A distinct Lewis structure representation is obviously needed to inform that the neutral Mo(N^t^BuAr)_3_ and Ti(N^t^BuPh)_3_ fragments provide different numbers of π electrons to the MoNNTi core, 3e^–^ and 1e^–^, respectively. We propose that this can be done by adding a positive and negative charge to the Mo and Ti centers. Within the ionic ligand formalism, the given Lewis representation implies an [N_2_]^4–^ ligand and Mo^VI^–Ti^IV^ oxidation states. Consistent with these assignments, the Mo–N bond distance of 1.79 Å [**5-**MoTi](μ-N_2_) is essentially equal to that of the isoelectronic dicationic dimolybdenum [**5-**Mo]_2_(μ-N_2_)^2+^ (1.80 Å).

## The Special Case of Metallocenes

Finally, we turn to the metallocenes of group 4 transition metals, which have a rich history in nitrogen fixation and exemplify some of the interesting features associated with geometric changes.^105−108^ We focus on the end-on bridging titanium complexes [Cp′_2_Ti]_2_(μ-N_2_) (Cp′ = substituted cyclopentadienyl). Depending on the substituents, these are known to have two limiting conformations in the solid state differentiated by whether the two Cp′ rings on one metal are staggered (*D*_2*d*_) or eclipsed (*D*_2*h*_) with respect to the Cp′ rings on the other metal.

**Scheme 3:**
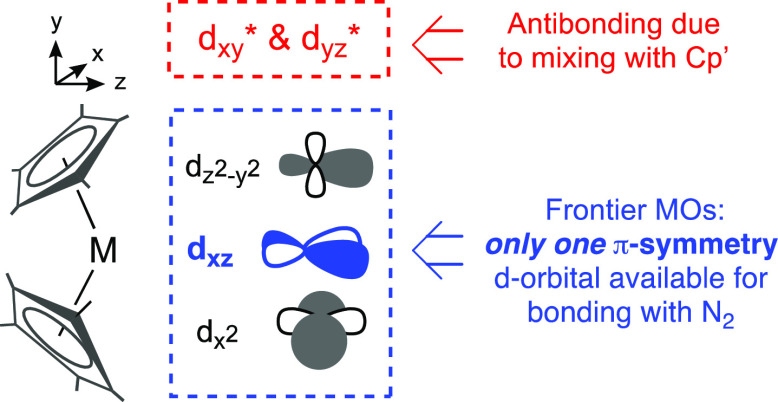
Frontier MOs in a Bent Metallocene Adapted from ref (109).

**Figure 10:**
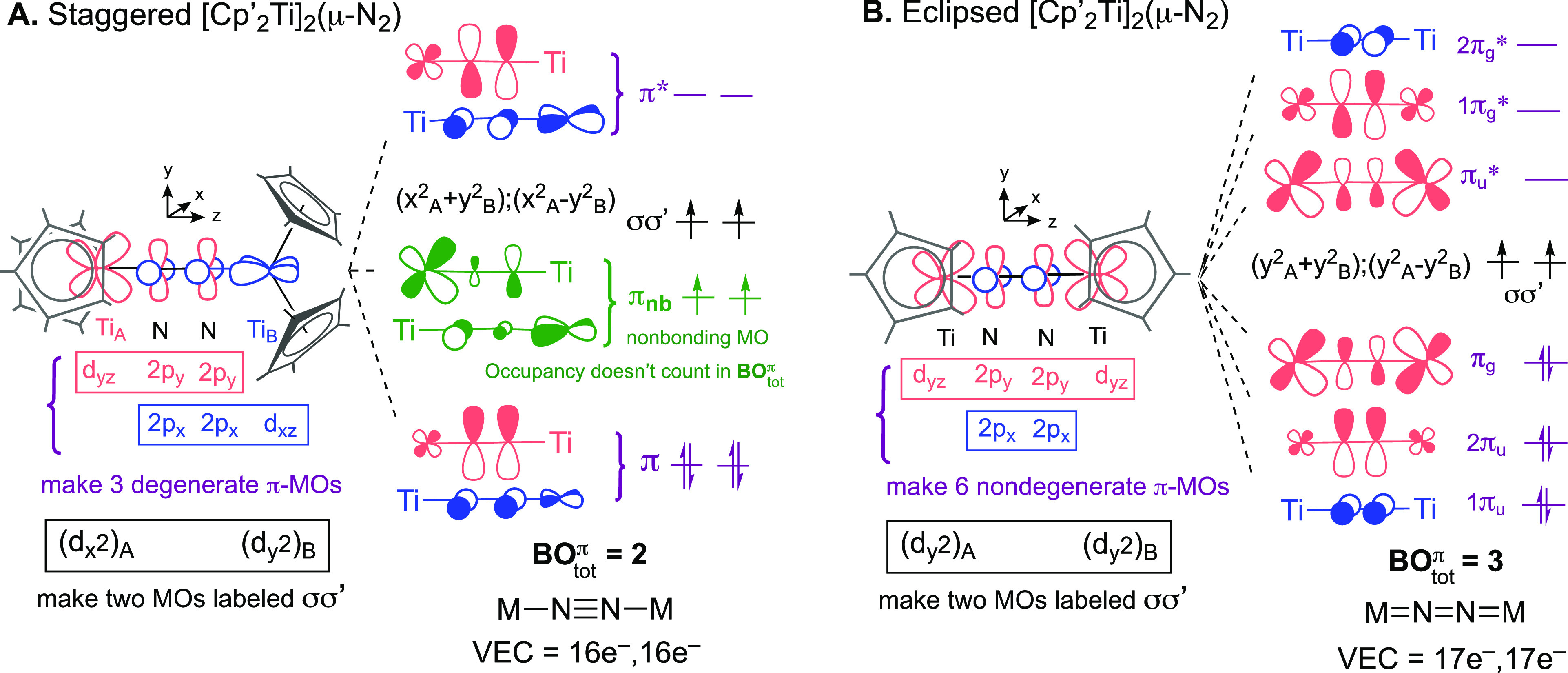
π-MO diagrams for staggered and eclipsed [Cp′_2_Ti]_2_(μ-N_2_).

In a bent metallocene fragment [Cp′_2_M], the d_*xy*_,d_*yz*_ AOs of the metal are used in bonding with the two η^5^-Cp′ rings.^109^ The remaining three metal d-AOs have their maximum probability density in the *xz* plane with largely d_*z*^2^–__*y*^2^_, d_*x*^2^_, and d_*xz*_ character as depicted in Scheme 3.^109^ There is therefore *only one* d-orbital on each metal available for π-bonding in μ-N_2_ metallocenes. This leads to MO diagrams that are dependent on the conformation as described in Figure 10.

The equivalent metals in the staggered [Cp′_2_Ti]_2_(μ-N_2_) in Figure 10A are labeled Ti_A_ and Ti_B_, and their Cp′ ligands are positioned on the *x* and *y* axes, respectively. In this case, d_*yz*_ from Ti_A_, d_*xz*_ from Ti_B_, and the four (2p_*x*_,2p_*y*_) AOs from N_2_ generate three doubly degenerate π-MOs: one bonding (π), one nonbonding (π_nb_), and one antibonding (π*). The d_*x*^2^_ on Ti_A_ and d_*y*^2^_ on Ti_B_ combine into two nonbonding MOs that we label as σσ′, like the δδ′ labels used in Figure 4. Note that the d_*z*^2^–*y*^2^_ AO shown in Scheme 3 is used for σ-bonding with N_2_; the resulting σ-MOs are not included in the final MO diagram. Because the opposing metal d-orbitals in Figure 10A are orthogonal to each other, there are two distinct π-systems that are degenerate, and the given picture of *bridging* π-bonding in Figure 10A is similar to the one in the *terminal* N_2_ complexes in Figure 2 above: the degree of occupancy of the nonbonding π-MO does not influence BO_tot_^π^ and the Lewis structure. The nonbonding character of both σσ’ and π_nb_ as obtained from the simplified MO diagram suggests μ-N_2_ metallocenes have accessible open shell ground state configurations and BO^π^ = 2 implying Ti–N≡N–Ti Lewis structure and a VEC = 16e^–^. This result aligns with the spectroscopic and MO analysis of [Cp*_2_Ti]_2_(μ-N_2_) reported by Bercaw and co-workers, including the presence of unpaired electrons.^110^ If the valence π-MOs were bonding in character, as in the nonmetallocenes, they would have been greatly stabilized relative to σσ′ leading to a closed shell ground state.

Drawing the staggered end-on N_2_-bridged metallocenes as M–N≡N–M is in accord with the short N–N distances crystallographically determined for [Cp*_2_Ti]_2_(μ-N_2_) (1.15 or 1.16 Å, depending on the unit cell). The formulation also accommodates the following reactivity observations: (i) Ti and Zr [Cp′_2_M]_2_(μ-N_2_) complexes react with N_2_ to form bridging [Cp′_2_M(N_2_)]_2_(μ-N_2_) as well as terminal Cp′_2_M(N_2_)_2_ products, both of which are diamagnetic 18e^–^ species;^111^ (ii) N_2_ substitution is often facile in metallocenes, implicating relatively weak M–N bonds; and (iii) no examples of direct thermal or photolytic N_2_ splitting to terminal nitride complexes have been reported. Interestingly, a different outcome is obtained for the eclipsed conformer, which is observed for several metallocenes such as [(C_5_Me_4_H)_2_Ti]_2_(μ-N_2_).^112^ The two metal d_*yz*_ AOs and the two 2p_*y*_ AOs of N_2_ are now all coplanar, transforming into two bonding and two antibonding nondegenerate π-MOs (Figure 10B). An additional pair of π and π* MOs is generated from the nitrogen 2p_*x*_ AOs which are orthogonal to the π-symmetry d-orbitals. According to this analysis, the eclipsed conformer has three π-bonds with a Ti=N=N=Ti Lewis structure. Note that, whereas staggered complexes [Cp′_2_M(N_2_)]_2_(μ-N_2_) are common, we are not aware of any complexes of this formula that adopt an eclipsed geometry, which would lead to a 19e^–^ VEC for each metal center. The modifications in the π-MO diagrams discussed in this section are also relevant when one metallocene is bridged by N_2_ to a nonmetallocene.^102,113^

## Conclusions and Outlook

Bridging N_2_ transition metal complexes have a vast and intricate chemistry, yet despite decades of intensive research, the principles that dictate their structure and reactivity are still being elucidated. Bonding in the linear MNNM core of these complexes leads to delocalized π-MOs with different numbers of nodes that define local MN and NN π-bond orders analogously to linear organic molecules. Such π-MO diagrams were used by Chatt and Richard in 1971,^114^ and in numerous subsequent studies to rationalize observed trends in the NN bond lengths and stretching vibrational frequencies.^115−117^ However, the generally accepted approach to assigning Lewis structures for the MNNM cores has been based on empirical comparisons of the bond length or vibrational frequency of the bridging N_2_ with those of free diazene or hydrazine. We note that the N–N bonds in linear MNNM cores are not comparable to the N–N single bond in hydrazine or the N=N double bond in diazene, just as the C–C bonds in diacetylene or ethylenedione are not comparable to those of ethane or ethylene. Instead of relying on this imperfect comparison to assign Lewis structures, we introduce an alternative model to assign Lewis structures based on qualitative π-MO diagrams. The model affords three limiting Lewis structures depending on the occupancy of the MOs: M–N≡N–M, M=N=N=M, and M≡N–N≡M.

The MO-based Lewis structures lead to a new classification of μ-N_2_ complexes that we hope will enable meaningful comparisons across the field for the first time. With this new classification system, for example, we can now assign reliable electron donor numbers to the μ-N_2_ ligand and engage in familiar valence electron- and bond-counting exercises to aid understanding and predict reactivity trends of μ-N_2_ complexes. This was illustrated for several important reactions of bridging N_2_ complexes: reaction with N_2_ to give two terminal MN_2_ products; reaction with H_2_ to give the corresponding NH_3_ adducts; and the splitting of MNNM into two metal nitrides. The MO-based model is broadly applicable across the periodic table and accommodates a wide variety of structural variations (e.g., coordination number, metallocenes, and heterobimetallic complexes). Computational DFT and *ab initio* methods will always be needed for quantitative purposes.^118^ However, the simple tools of tracking the number of bonds and VECs which are possible only with reliable Lewis structures can help advance the field by providing an intuitive understanding of bonding and reactivity patterns and providing a tool for “back of the envelope” predictions that can help rationalize reaction outcomes and guide the design of new catalysts.

## Supporting Information Available

Figures for MO displays and qualitative MO diagrams. Cartesian coordinates and absolute energies of the new molecules used to calculate the hydrogenation energies in Figure 5 (PDF)
