# Supplementary material for: Lewis Structures and the Bonding Classification of End-on Bridging Dinitrogen Transition Metal Complexes
Source: J Am Chem Soc. 2023 Feb 16;145(8):4326–42. doi: 10.1021/jacs.2c12243 (PMC9983020; doi:10.1021/jacs.2c12243)
Supplement: Supplementary file 1 — ja2c12243_si_001.pdf [file ja2c12243_si_001.pdf]

Supporting Information for:

Lewis Structures and the Bonding Classification of End-on Bridging Dinitrogen Transition Metal Complexes

Faraj Hasanayn,[1,*] Patrick L. Holland,[2] Alan S. Goldman,[3] Alexander J. M. Miller[4,*]

[1] Department of Chemistry, *American University of Beirut*, Beirut 1107 2020, Lebanon

[2] Department of Chemistry, *Yale University*, New Haven, Connecticut 06520, United States

[3] Department of Chemistry and Chemical Biology, *Rutgers, The State University of New Jersey*, New Brunswick, New Jersey 08903, United States

[4] Department of Chemistry, *University of North Carolina at Chapel Hill*, Chapel Hill, North Carolina 27599-3290, United States

Corresponding Authors

*E-mail: fh19@aub.edu.lb (F.H.)

*E-mail: ajmm@email.unc.edu (A.J.M.M.)

Table of Contents

Figure SI-1: Using a fragment approach to construct the π-MOs in $[N_3]^-$ and cyanogen. (Page SI2)

Figure SI-2: Kohn-Sham π-MOs for $[Ru(NH_3)_5(NN)]^{2+}$ and [**1**-Re]$_2$(μ-N_2). (Page SI3)

Figure SI-3: Kohn-Sham π-MOs for CNNC, OCCO and FCCF (**C**). (Page SI3)

Figure SI-4: Qualitative MO diagram for μ-N_2 complexes in eclipsed octahedral, square pyramidal and trigonal bipyramidal geometries. (Page SI4)

Figure SI-5: Qualitative MO diagrams for μ-N_2 complexes in staggered trigonal bipyramidal and tetragonal geometries. (Page SI4)

Figure SI-6: Qualitative MO diagrams for μ-N_2 complexes in staggered square planar and staggered and eclipsed trigonal planar geometries. (Page SI5)

Table S1: Cartesian Coordinates and Absolute Energies of the Species Used in Hydrogenation of the Bridging N_2 with H_2. (Page SI6)

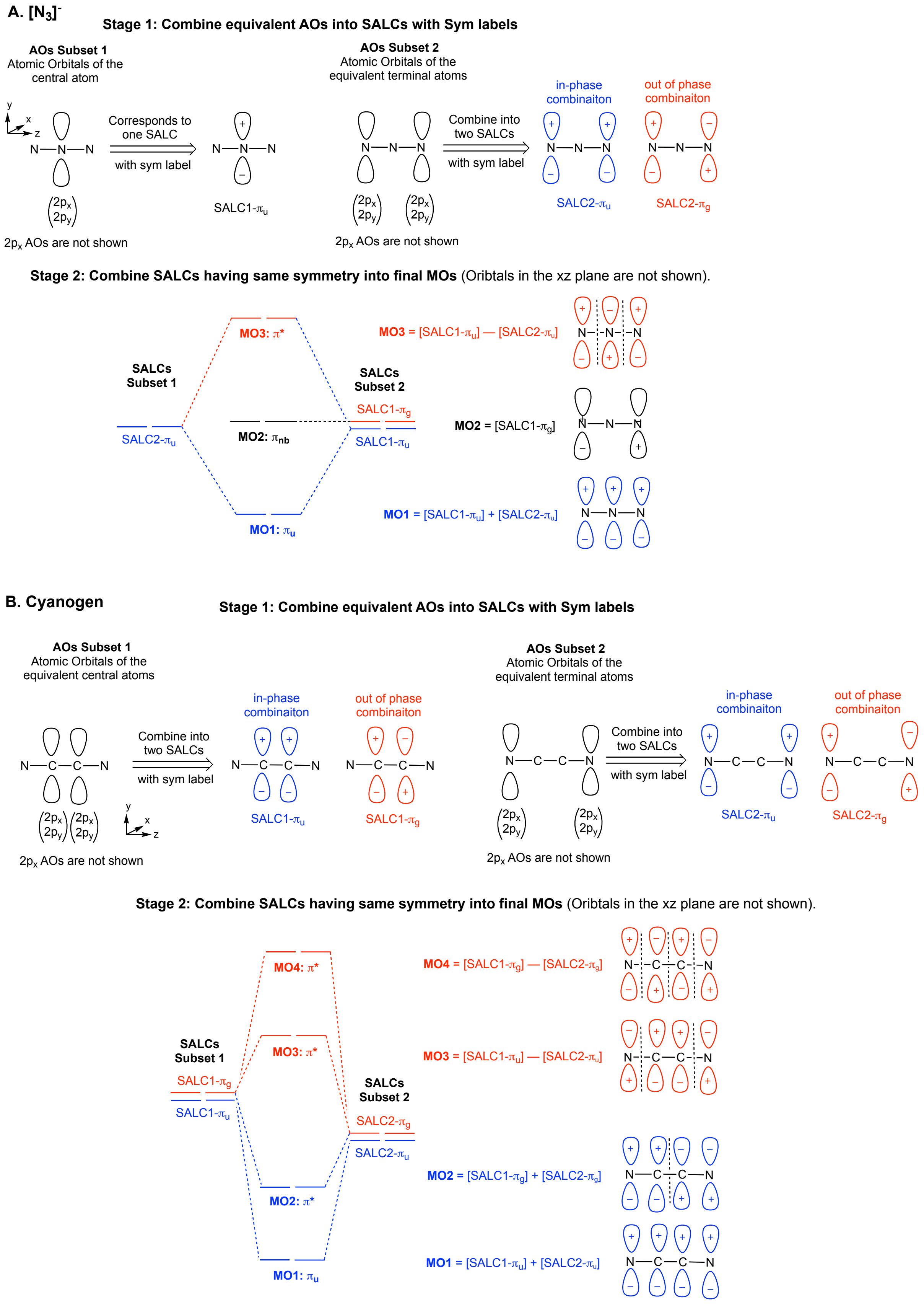

Figure SI-1: Using a fragment approach to construct the π-MOs in $[N_3]^-$ and cyanogen.

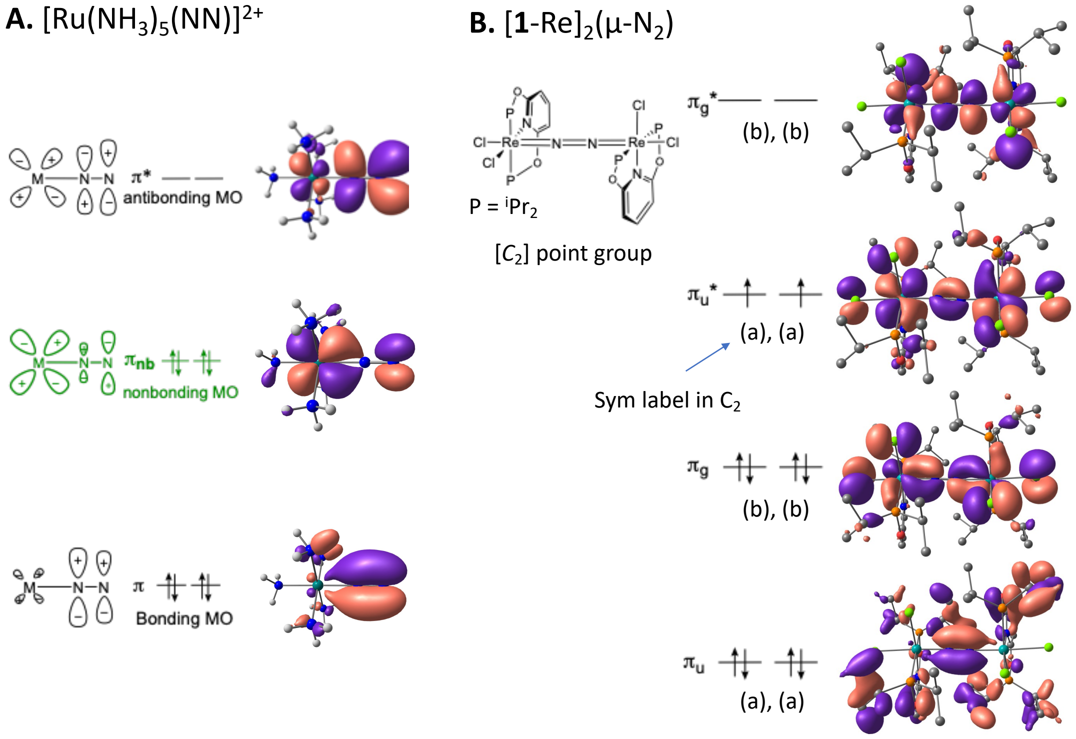

Figure SI-2: Kohn-Sham π-MOs for $[Ru(NH_3)_5(NN)]^{2+}$ (**A**), and $[$**1**$\text{-Re}]_2(\mu\text{-}N_2)$ (**B**) computed using the M06L functional using an isosurface of 0.03 eÅ^{-3}. Only one of each pair of similar MOs is shown.

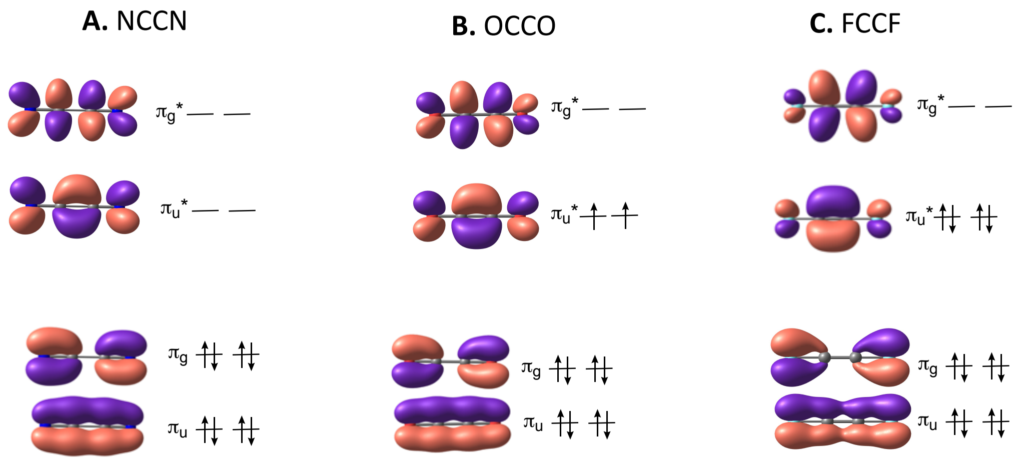

Figure SI-3: Kohn-Sham π-MOs for CNNC (**A**), OCC (**B**) and FCCF (**C**) computed using the M06L functional and an isosurface of 0.06 eÅ^{-3}. Only one of each pair of degenerate MOs is shown.

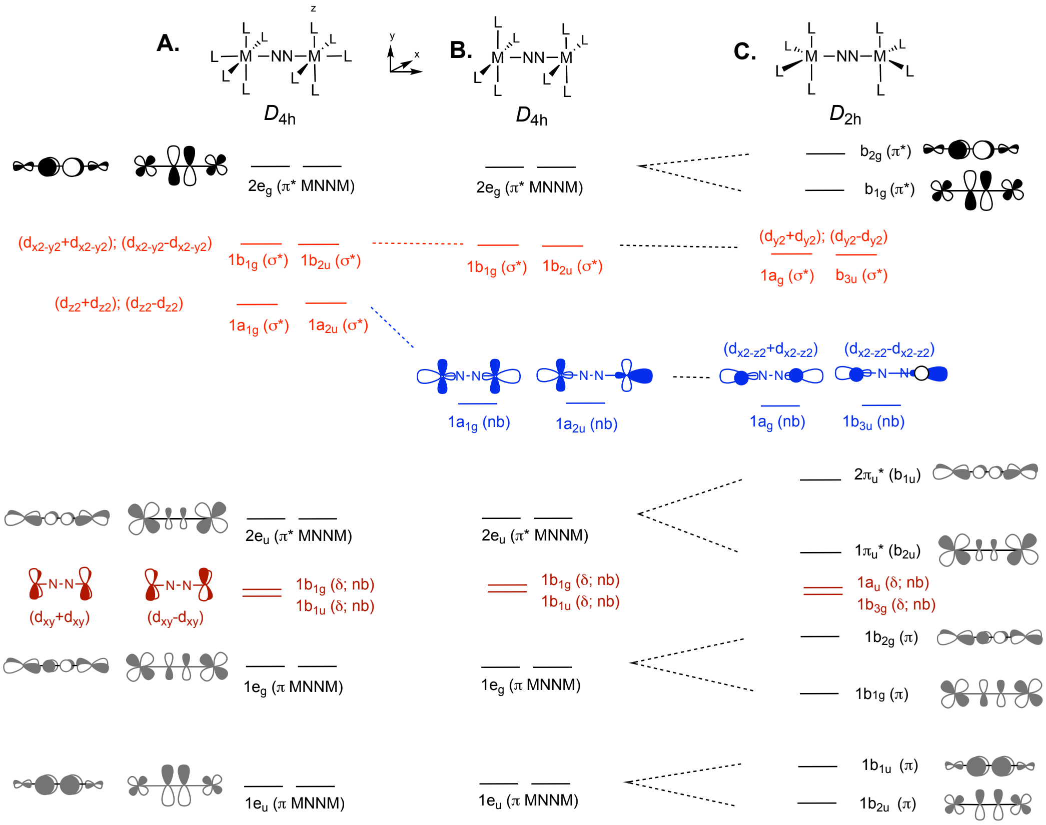

Figure SI-4: Qualitative MO diagram for μ-N_2 complexes in eclipsed octahedral (**A**), square pyramidal (**B**) and trigonal bipyramidal (**C**) geometries. Energy order is arbitrary.

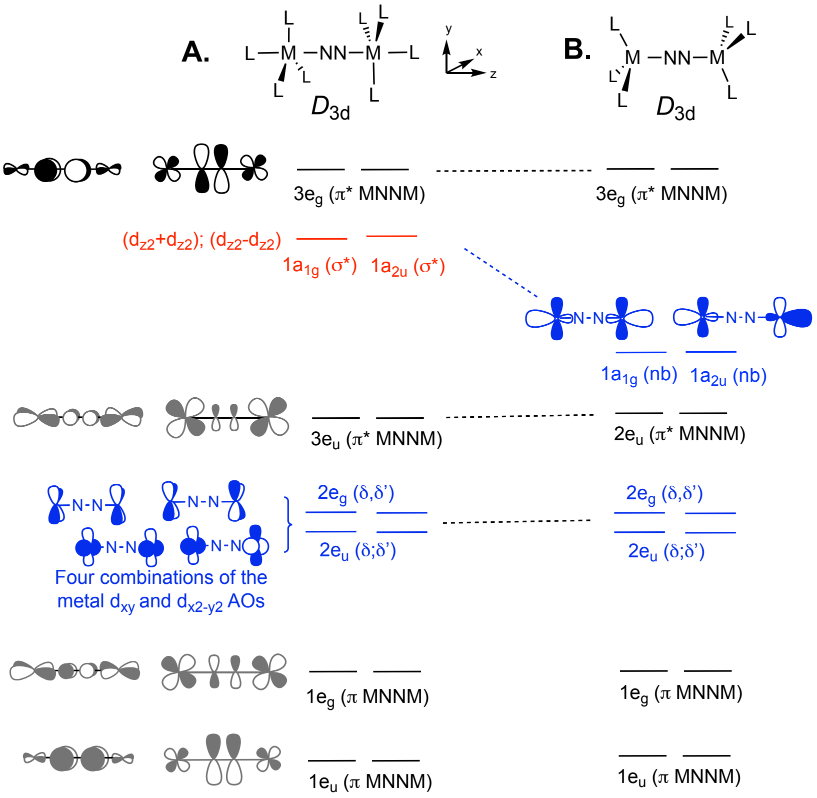

Figure SI-5: Qualitative MO diagrams for μ-N_2 complexes in staggered trigonal bipyramidal and tetragonal geometries. Energy order is arbitrary.

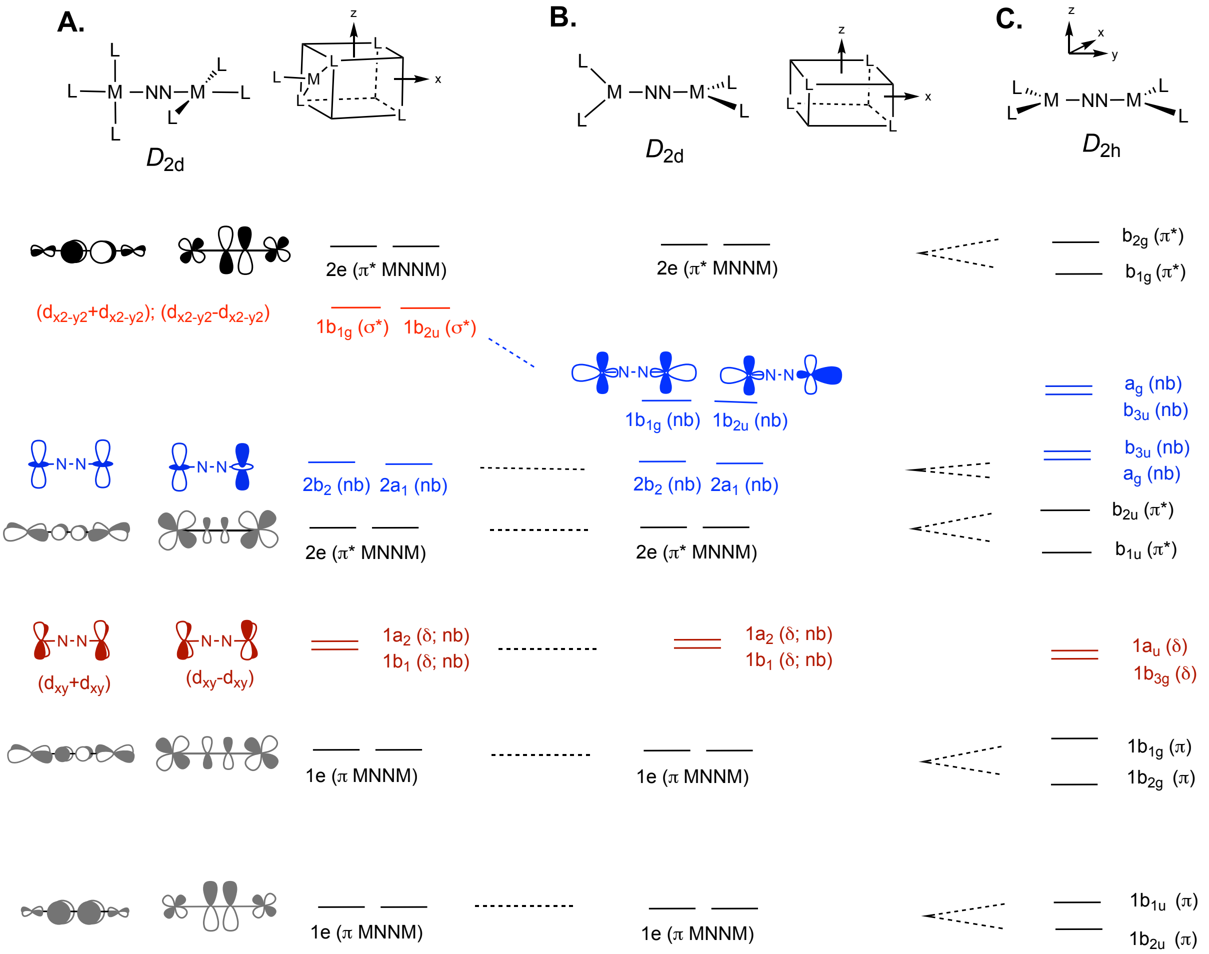

Figure SI-6: Qualitative MO diagrams for μ-N_2 complexes in staggered square planar and staggered and eclipsed trigonal planar geometries.

Table 1: Cartesian Coordinates and Absolute Energies of the Species Used in Hydrogenation of the Bridging N2 with H2.

114

[(PONOP)W(Cl)2]2(N2) E(def2) = -5194.490142 au. Correction to G = 0.832725 au.

W	2.415700	0.380500	-0.383000
N	0.604900	0.131100	-0.272700
N	-0.604900	-0.131100	-0.272700
N	2.832300	-1.004800	1.281700
C	2.926600	-2.343300	1.055900
C	3.074700	-0.533600	2.535000
C	3.236100	-3.242700	2.072500
C	3.388100	-1.380100	3.593600
C	3.468500	-2.740600	3.342800
H	3.304700	-4.296100	1.837100
H	3.573100	-0.954000	4.569900
H	3.722600	-3.420000	4.149800
O	2.713400	-2.824500	-0.164300
O	3.017600	0.774700	2.776300
P	2.610200	-1.739900	-1.547900
P	2.661200	1.904200	1.475500
C	4.032900	-2.376000	-2.553500
C	1.194000	2.769600	2.199500
C	1.097000	-2.434800	-2.325300
C	4.125300	2.995200	1.740000
H	3.620400	-3.214700	-3.130900
H	1.575500	3.458300	2.963200
H	0.279000	-2.078400	-1.689000
H	4.953500	2.298800	1.564700
W	-2.415700	-0.380600	-0.383000
N	-2.832300	1.004900	1.281600
C	-2.926600	2.343300	1.055800
C	-3.074700	0.533600	2.534900
C	-3.236200	3.242800	2.072300
C	-3.388100	1.380200	3.593600
C	-3.468500	2.740700	3.342700
H	-3.304700	4.296100	1.836900
H	-3.573100	0.954100	4.569800
H	-3.722600	3.420200	4.149700
O	-2.713400	2.824500	-0.164400
O	-3.017600	-0.774700	2.776300
P	-2.610200	1.739900	-1.548000
P	-2.661200	-1.904100	1.475500
C	-4.032900	2.375900	-2.553600
C	-1.194100	-2.769500	2.199600
C	-1.097000	2.434700	-2.325400
C	-4.125300	-2.995200	1.740100
H	-3.620400	3.214600	-3.131000

H	-1.575500	-3.458200	2.963400
H	-0.279000	2.078300	-1.689100
H	-4.953500	-2.298800	1.564800
C	0.925600	-1.782000	-3.696300
H	1.653900	-2.168400	-4.417300
H	-0.076200	-1.997900	-4.075800
H	1.038300	-0.693700	-3.649300
C	1.031300	-3.953700	-2.388200
H	0.041700	-4.250900	-2.747200
H	1.770600	-4.369000	-3.080300
H	1.186500	-4.414200	-1.410500
C	-0.220500	-1.795700	2.846600
H	0.634400	-2.343400	3.255000
H	0.169700	-1.090000	2.107400
H	-0.676600	-1.224100	3.657400
C	-0.510700	-3.558000	1.083200
H	-0.101300	-2.873800	0.333600
H	0.319900	-4.145900	1.487400
H	-1.184900	-4.239000	0.557500
C	-0.925500	1.781900	-3.696300
H	-1.653900	2.168300	-4.417400
H	0.076200	1.997700	-4.075800
H	-1.038300	0.693600	-3.649400
C	-1.031300	3.953600	-2.388300
H	-0.041600	4.250800	-2.747400
H	-1.770600	4.368900	-3.080500
H	-1.186500	4.414200	-1.410600
C	0.510700	3.558000	1.083100
H	0.101300	2.873800	0.333500
H	-0.319900	4.145900	1.487300
H	1.184900	4.239000	0.557400
C	0.220500	1.795800	2.846600
H	-0.634400	2.343500	3.254900
H	-0.169700	1.090000	2.107400
H	0.676600	1.224200	3.657300
C	5.192900	-2.889300	-1.708200
H	5.587200	-2.104900	-1.057800
H	4.903800	-3.739200	-1.087500
H	6.004800	-3.213100	-2.366200
C	4.469200	-1.274500	-3.520600
H	4.914100	-0.435700	-2.981200
H	5.214200	-1.670000	-4.217400
H	3.639300	-0.876500	-4.109300
C	4.243700	3.573900	3.142300
H	5.219800	4.052900	3.263100
H	3.489700	4.344100	3.331500
H	4.152600	2.807600	3.915000

C	4.163400	4.061100	0.648300
H	3.373500	4.806700	0.785300
H	5.117300	4.594400	0.683400
H	4.054200	3.627400	-0.349800
C	-4.163400	-4.061000	0.648400
H	-3.373500	-4.806600	0.785500
H	-5.117300	-4.594400	0.683600
H	-4.054200	-3.627400	-0.349700
C	-4.243700	-3.573800	3.142400
H	-5.219800	-4.052800	3.263200
H	-3.489700	-4.344000	3.331600
H	-4.152600	-2.807400	3.915100
C	-5.192800	2.889200	-1.708400
H	-5.587200	2.104900	-1.057900
H	-4.903800	3.739200	-1.087700
H	-6.004800	3.213000	-2.366300
C	-4.469200	1.274400	-3.520700
H	-4.914100	0.435600	-2.981300
H	-5.214100	1.669800	-4.217500
H	-3.639300	0.876300	-4.109300
Cl	4.899300	0.457800	-0.381600
Cl	2.377200	1.985800	-2.267000
Cl	-4.899300	-0.457800	-0.381600
Cl	-2.377200	-1.985900	-2.267000

114

[(PONOP)Re(Cl)2]2(N2) E(def2) = -5216.90594 au. Correction to G = 0.835624 au.

Re	-0.345900	2.469900	-0.328000
N	-0.105600	0.577800	-0.257700
N	0.105600	-0.577800	-0.257700
N	1.002900	2.849400	1.276400
C	2.335200	2.993900	1.036800
C	0.536200	3.067100	2.536700
C	3.232100	3.328300	2.045000
C	1.380300	3.409900	3.585600
C	2.735500	3.539500	3.322300
H	4.279900	3.434100	1.798900
H	0.957000	3.576300	4.566400
H	3.413600	3.815000	4.123000
O	2.799300	2.802100	-0.195700
O	-0.769100	2.944100	2.780400
P	1.686800	2.653300	-1.534400
P	-1.867700	2.607700	1.465900
C	2.235700	4.073000	-2.591500
C	-2.756700	1.137100	2.151800
C	2.380800	1.157600	-2.337500
C	-2.988100	4.051800	1.704000
H	3.062300	3.672300	-3.194400

H	-3.483300	1.511100	2.883100
H	2.057600	0.327500	-1.700100
H	-2.301200	4.891500	1.549700
Re	0.345900	-2.469900	-0.328000
N	-1.002900	-2.849400	1.276400
C	-2.335200	-2.993900	1.036800
C	-0.536200	-3.067100	2.536700
C	-3.232100	-3.328300	2.045000
C	-1.380300	-3.409900	3.585600
C	-2.735500	-3.539500	3.322300
H	-4.279900	-3.434100	1.798900
H	-0.957000	-3.576300	4.566400
H	-3.413600	-3.815000	4.123000
O	-2.799300	-2.802100	-0.195700
O	0.769100	-2.944100	2.780400
P	-1.686800	-2.653300	-1.534400
P	1.867700	-2.607700	1.465900
C	-2.235700	-4.073000	-2.591500
C	2.756700	-1.137100	2.151800
C	-2.380800	-1.157600	-2.337500
C	2.988100	-4.051800	1.704000
H	-3.062300	-3.672300	-3.194400
H	3.483300	-1.511100	2.883100
H	-2.057600	-0.327500	-1.700100
H	2.301200	-4.891500	1.549700
C	1.689100	0.979500	-3.688400
H	2.052000	1.705000	-4.424000
H	1.897700	-0.024100	-4.067600
H	0.601700	1.087600	-3.610100
C	3.897600	1.113700	-2.443700
H	4.197600	0.130700	-2.818900
H	4.284200	1.864200	-3.140400
H	4.383000	1.266600	-1.477600
C	1.809700	-0.167000	2.842300
H	2.371800	0.692800	3.219500
H	1.066100	0.216600	2.138400
H	1.280600	-0.622600	3.681500
C	3.490300	-0.451200	1.000100
H	2.771900	-0.042000	0.283700
H	4.095300	0.379600	1.377500
H	4.146500	-1.122600	0.440700
C	-1.689100	-0.979500	-3.688400
H	-2.052000	-1.705000	-4.424000
H	-1.897700	0.024100	-4.067600
H	-0.601700	-1.087600	-3.610100
C	-3.897600	-1.113700	-2.443700
H	-4.197600	-0.130700	-2.818900

H	-4.284200	-1.864200	-3.140400
H	-4.383000	-1.266600	-1.477600
C	-3.490300	0.451200	1.000100
H	-2.771900	0.042000	0.283700
H	-4.095300	-0.379600	1.377500
H	-4.146500	1.122600	0.440700
C	-1.809700	0.167000	2.842300
H	-2.371800	-0.692800	3.219500
H	-1.066100	-0.216600	2.138400
H	-1.280600	0.622600	3.681500
C	2.750000	5.261900	-1.788000
H	1.980500	5.647100	-1.115100
H	3.631500	5.007500	-1.196600
H	3.026300	6.068600	-2.473500
C	1.085800	4.460300	-3.523200
H	0.258500	4.896400	-2.960100
H	1.437100	5.198400	-4.250300
H	0.685500	3.609000	-4.078400
C	-3.606200	4.156600	3.090600
H	-4.091000	5.130600	3.204700
H	-4.379200	3.399600	3.254100
H	-2.862000	4.063300	3.884800
C	-4.023100	4.080300	0.582500
H	-4.768500	3.287100	0.701400
H	-4.561400	5.031800	0.599800
H	-3.559900	3.971600	-0.402300
C	4.023100	-4.080300	0.582500
H	4.768500	-3.287100	0.701400
H	4.561400	-5.031800	0.599800
H	3.559900	-3.971600	-0.402300
C	3.606200	-4.156600	3.090600
H	4.091000	-5.130600	3.204700
H	4.379200	-3.399600	3.254100
H	2.862000	-4.063300	3.884800
C	-2.750000	-5.261900	-1.788000
H	-1.980500	-5.647100	-1.115100
H	-3.631500	-5.007500	-1.196600
H	-3.026300	-6.068600	-2.473500
C	-1.085800	-4.460300	-3.523200
H	-0.258500	-4.896400	-2.960100
H	-1.437100	-5.198400	-4.250300
H	-0.685500	-3.609000	-4.078400
Cl	-0.536200	4.919900	-0.392100
Cl	-1.935600	2.288500	-2.203900
Cl	0.536200	-4.919900	-0.392100
Cl	1.935600	-2.288500	-2.203900

114

[(PONOP)Os(Cl)2]2(N2) E(def2) = -5241.737821 au. Correction to 0.839194 au.

Os	-2.495800	-0.349700	-0.291100
N	-0.563900	-0.095800	-0.251900
N	0.563800	0.095700	-0.251900
N	-2.897300	0.962700	1.275000
C	-3.097200	2.283200	1.029400
C	-3.091700	0.494400	2.534700
C	-3.462700	3.171500	2.032700
C	-3.464400	1.326500	3.581300
C	-3.649200	2.675100	3.314300
H	-3.610900	4.212600	1.780000
H	-3.609500	0.899300	4.563800
H	-3.948500	3.345200	4.112700
O	-2.923700	2.738800	-0.213300
O	-2.903100	-0.808300	2.774300
P	-2.723300	1.619000	-1.518500
P	-2.555800	-1.875300	1.458300
C	-4.145400	2.070500	-2.613800
C	-1.063500	-2.749800	2.113300
C	-1.249500	2.334300	-2.338100
C	-3.964000	-3.042600	1.664500
H	-3.760400	2.890600	-3.236200
H	-1.423700	-3.498600	2.828700
H	-0.405500	2.051500	-1.700200
H	-4.822200	-2.375600	1.527700
Os	2.495800	0.349700	-0.291200
N	2.897300	-0.962700	1.275000
C	3.097200	-2.283200	1.029500
C	3.091700	-0.494300	2.534700
C	3.462800	-3.171400	2.032800
C	3.464500	-1.326300	3.581300
C	3.649300	-2.674900	3.314500
H	3.611000	-4.212500	1.780200
H	3.609600	-0.899100	4.563900
H	3.948700	-3.345000	4.112800
O	2.923600	-2.738800	-0.213200
O	2.903000	0.808400	2.774300
P	2.723200	-1.619100	-1.518400
P	2.555800	1.875400	1.458200
C	4.145400	-2.070700	-2.613600
C	1.063600	2.750100	2.113100
C	1.249500	-2.334400	-2.338000
C	3.964000	3.042600	1.664400
H	3.760400	-2.890800	-3.236100
H	1.424000	3.499100	2.828300
H	0.405500	-2.051500	-1.700100
H	4.822300	2.375600	1.527500

C	-1.049000	1.622600	-3.675300
H	-1.779200	1.953600	-4.420900
H	-0.048200	1.849500	-4.051800
H	-1.129900	0.534300	-3.577400
C	-1.250800	3.849800	-2.472400
H	-0.278900	4.170600	-2.859000
H	-2.016200	4.202000	-3.171100
H	-1.411800	4.348500	-1.514400
C	0.103600	1.809100	2.825600
H	-0.761000	2.371700	3.191400
H	-0.276000	1.046600	2.140800
H	0.565100	1.302600	3.675500
C	0.370100	3.450400	0.945100
H	-0.037700	2.713200	0.248200
H	-0.463300	4.058900	1.310700
H	1.034600	4.096400	0.366100
C	1.049000	-1.622800	-3.675200
H	1.779200	-1.953900	-4.420800
H	0.048200	-1.849700	-4.051800
H	1.130000	-0.534500	-3.577400
C	1.250800	-3.849900	-2.472200
H	0.278800	-4.170700	-2.858700
H	2.016200	-4.202200	-3.170800
H	1.411700	-4.348500	-1.514100
C	-0.370100	-3.450400	0.945400
H	0.037500	-2.713300	0.248200
H	0.463400	-4.058600	1.311000
H	-1.034600	-4.096600	0.366700
C	-0.103500	-1.808400	2.825300
H	0.761300	-2.370700	3.191000
H	0.275700	-1.045900	2.140200
H	-0.564900	-1.301800	3.675200
C	-5.363600	2.573200	-1.847200
H	-5.741300	1.810300	-1.163300
H	-5.144200	3.476100	-1.273900
H	-6.161400	2.811600	-2.556900
C	-4.477800	0.881300	-3.516900
H	-4.894400	0.056600	-2.936300
H	-5.214900	1.188800	-4.264600
H	-3.604900	0.494700	-4.047400
C	-4.046100	-3.694800	3.037400
H	-5.009700	-4.201100	3.144600
H	-3.273300	-4.456400	3.178900
H	-3.964100	-2.968200	3.849300
C	-3.970900	-4.051300	0.519000
H	-3.162100	-4.782400	0.619600
H	-4.911200	-4.609200	0.526400

H	-3.876000	-3.562000	-0.454500
C	3.970900	4.051400	0.519000
H	3.162000	4.782500	0.619600
H	4.911100	4.609300	0.526300
H	3.875900	3.562100	-0.454600
C	4.046300	3.694700	3.037300
H	5.009900	4.201100	3.144400
H	3.273500	4.456300	3.178900
H	3.964500	2.968100	3.849100
C	5.363600	-2.573400	-1.847000
H	5.741300	-1.810500	-1.163200
H	5.144200	-3.476200	-1.273800
H	6.161300	-2.811800	-2.556700
C	4.477800	-0.881500	-3.516800
H	4.894400	-0.056800	-2.936300
H	5.214900	-1.189000	-4.264500
H	3.604900	-0.494900	-4.047300
Cl	-4.925100	-0.638600	-0.382100
Cl	-2.227900	-1.929000	-2.162500
Cl	4.925100	0.638500	-0.382200
Cl	2.227900	1.928900	-2.162600

60

[(PONOP)W(Cl)2(NH3)] E(def2) = -2598.997332 au. Correction to G = 0.432251 au.

W	-0.000100	-0.529900	0.033600
Cl	0.000300	-3.023900	-0.233400
Cl	-0.000000	-0.157300	-2.383100
N	-0.000000	-1.234800	2.239400
N	-0.000000	1.660100	0.214700
C	1.165200	2.372800	0.190400
C	-1.165200	2.372800	0.190000
C	1.200700	3.759900	0.138600
C	-1.200700	3.759900	0.138300
C	-0.000000	4.451800	0.109400
H	0.000000	5.535200	0.060200
H	2.161700	4.255300	0.113700
H	-2.161600	4.255300	0.113100
O	2.328500	1.721300	0.240500
O	-2.328500	1.721300	0.239700
P	2.330500	-0.026500	0.191800
P	-2.330300	-0.026800	0.191800
C	3.541100	-0.345900	-1.183400
C	3.255000	-1.728600	-1.768100
C	3.507700	0.743700	-2.247900
C	-3.343600	-0.253300	1.735600
C	-4.561600	0.653200	1.852100
C	-3.708800	-1.730400	1.862900
C	3.343700	-0.253900	1.735500

C	4.561900	0.652300	1.852400
C	3.708500	-1.731100	1.862300
C	-3.541100	-0.346700	-1.183200
C	-3.254800	-1.729500	-1.767600
C	-3.508100	0.742700	-2.248000
H	2.509400	0.835200	-2.683600
H	3.799900	1.716800	-1.849400
H	4.199600	0.484100	-3.054900
H	2.281600	-1.748000	-2.264100
H	4.022400	-1.984700	-2.505100
H	3.241100	-2.515100	-1.008700
H	-4.448800	-2.014400	1.108000
H	-2.845700	-2.390800	1.728400
H	-4.152700	-1.939100	2.840600
H	-5.301000	0.435800	1.076000
H	-5.057500	0.498900	2.815700
H	-4.293400	1.707200	1.773200
H	4.293900	1.706400	1.773500
H	5.057600	0.497800	2.816100
H	5.301300	0.434800	1.076400
H	4.152300	-1.940300	2.840000
H	2.845300	-2.391300	1.727400
H	4.448500	-2.015000	1.107400
H	-2.281400	-1.748900	-2.263600
H	-3.240700	-2.515700	-1.008000
H	-4.022200	-1.985900	-2.504500
H	-2.510000	0.834200	-2.684000
H	-4.200200	0.482700	-3.054700
H	-3.800500	1.715800	-1.849700
H	-4.535000	-0.355700	-0.715400
H	4.535000	-0.354800	-0.715800
H	-2.649800	0.016800	2.546000
H	2.649900	0.016100	2.545900
H	0.813500	-1.021900	2.811400
H	0.000100	-2.246900	2.079100
H	-0.813600	-1.022000	2.811300

60

[(PONOP)Re(Cl)2(NH3)] E(def2) = -2610.22626 au. Correction to G = 0.436 au.

Re	0.000000	-0.461500	-0.077900
Cl	0.020000	-2.936700	0.066200
Cl	-0.022300	-0.005200	2.341900
N	0.011400	-0.984500	-2.271700
N	-0.004800	1.637000	-0.335000
C	-1.177700	2.338100	-0.309300
C	1.169000	2.336400	-0.364400
C	-1.208500	3.726300	-0.355700
C	1.200400	3.724200	-0.411400

C	-0.004400	4.411300	-0.408500
H	-0.004100	5.495400	-0.436400
H	-2.165400	4.228900	-0.333200
H	2.158200	4.225100	-0.432600
O	-2.326500	1.672500	-0.250900
O	2.317600	1.667500	-0.362900
P	-2.279600	-0.079400	-0.106600
P	2.278900	-0.073800	-0.111800
C	-3.481500	-0.343300	1.286800
C	-3.135900	-1.676600	1.951900
C	-3.514900	0.809700	2.283000
C	3.333600	-0.503900	-1.581800
C	4.609000	0.312600	-1.742900
C	3.601200	-2.007200	-1.562500
C	-3.306500	-0.425500	-1.617900
C	-4.601200	0.368100	-1.732300
C	-3.533600	-1.932400	-1.713700
C	3.463600	-0.235200	1.312100
C	3.161700	-1.554600	2.024800
C	3.421600	0.955800	2.263000
H	-2.528400	0.979100	2.719700
H	-3.851100	1.739400	1.820400
H	-4.205400	0.567100	3.096800
H	-2.169600	-1.618600	2.456700
H	-3.901300	-1.930900	2.691600
H	-3.073400	-2.501300	1.236300
H	4.303300	-2.265800	-0.763700
H	2.690800	-2.592600	-1.394100
H	4.053200	-2.334100	-2.503600
H	5.334300	0.088600	-0.956200
H	5.092400	0.073700	-2.695700
H	4.412100	1.385600	-1.723500
H	-4.433200	1.440400	-1.619300
H	-5.063900	0.199200	-2.709800
H	-5.331800	0.060000	-0.979500
H	-3.977900	-2.201000	-2.676800
H	-2.608100	-2.505100	-1.585900
H	-4.226700	-2.269600	-0.936500
H	2.181700	-1.520100	2.504500
H	3.155900	-2.410700	1.344300
H	3.918800	-1.742400	2.792500
H	2.419800	1.092600	2.676200
H	4.107000	0.779200	3.097800
H	3.721800	1.882800	1.770500
H	4.467600	-0.294100	0.871900
H	-4.472500	-0.430600	0.822200
H	2.687400	-0.272700	-2.441500

H	-2.657900	-0.106000	-2.447600
H	-0.868200	-0.880300	-2.773000
H	0.202700	-1.988700	-2.227600
H	0.728800	-0.561200	-2.855300

60

[(PONOP)Os(Cl)2(NH3)] E(def2) = -2622.650966 au. Correction to G = 0.437096 au.

Os	0.000700	-0.426200	0.101700
Cl	-0.008000	-2.883900	-0.229600
Cl	0.001100	0.159300	-2.298900
N	-0.000200	-1.139500	2.177800
N	0.000700	1.626700	0.373500
C	1.165100	2.330800	0.360100
C	-1.163000	2.331400	0.361000
C	1.202800	3.716200	0.397800
C	-1.200100	3.716900	0.398800
C	0.001600	4.409500	0.421400
H	0.001900	5.493300	0.442200
H	2.164000	4.211600	0.390000
H	-2.161000	4.212700	0.391800
O	2.318500	1.649700	0.330100
O	-2.316900	1.650900	0.332300
P	2.263800	-0.061400	0.124000
P	-2.263500	-0.059600	0.124200
C	3.428200	-0.325000	-1.296700
C	3.035500	-1.621700	-2.005700
C	3.491500	0.864400	-2.248300
C	-3.286700	-0.520100	1.599300
C	-4.581600	0.266800	1.755500
C	-3.514300	-2.030100	1.582700
C	3.286200	-0.520800	1.600000
C	4.582400	0.264000	1.755300
C	3.511100	-2.031200	1.585900
C	-3.427400	-0.320700	-1.297300
C	-3.036400	-1.617300	-2.007400
C	-3.488300	0.869700	-2.247700
H	2.510600	1.071600	-2.680800
H	3.847000	1.768600	-1.750000
H	4.180700	0.635600	-3.066800
H	2.063100	-1.519200	-2.491200
H	3.783500	-1.865000	-2.766500
H	2.962400	-2.471500	-1.321400
H	-4.214200	-2.307400	0.788400
H	-2.590600	-2.592600	1.405800
H	-3.950700	-2.368400	2.526800
H	-5.295400	0.033100	0.961100
H	-5.066000	0.011700	2.703100
H	-4.406900	1.344000	1.744600

H	4.409700	1.341500	1.741300
H	5.065500	0.010600	2.703900
H	5.296400	0.026800	0.962100
H	3.946500	-2.368900	2.530700
H	2.586500	-2.592400	1.409500
H	4.210800	-2.311100	0.792300
H	-2.063300	-1.516000	-2.491900
H	-2.965400	-2.468000	-1.324000
H	-3.784100	-1.858200	-2.769200
H	-2.506800	1.075800	-2.679500
H	-4.177500	0.642800	-3.066900
H	-3.842700	1.774000	-1.748800
H	-4.419300	-0.455100	-0.845100
H	4.419600	-0.460500	-0.843800
H	-2.646200	-0.262100	2.455800
H	2.645800	-0.260100	2.455700
H	0.813400	-0.905300	2.740700
H	-0.002000	-2.158300	2.047200
H	-0.813200	-0.902700	2.740500

2

H2 molecules E(def2) = -1.171224063 au. Correction to G = -0.001598 au.

H	0.000000	0.000000	0.372279
H	0.000000	0.000000	-0.372279
